# Molecular characterization and prognostic modeling associated with M2-like tumor-associated macrophages in breast cancer: revealing the immunosuppressive role of DLG3

**DOI:** 10.3389/fimmu.2025.1650726

**Published:** 2025-08-13

**Authors:** Ziqiang Wang, Jing Zhang, Huili Chen, Xinyu Zhang, Kai Zhang, Feiyue Zhang, Yiluo Xie, Hongyu Ma, Linfeng Pan, Qiang Zhang, Min Lu, Hongtao Wang, Chaoqun Lian

**Affiliations:** ^1^ Key Laboratory of Cancer Research and Clinical Laboratory Diagnosis, Bengbu Medical University, Bengbu, China; ^2^ Department of Genetics, School of Life Sciences, Bengbu Medical University, Bengbu, China; ^3^ Department of Clinical Medicine, Bengbu Medical University, Bengbu, China; ^4^ Department of Urology, The First Affiliated Hospital of Xiamen University, School of Medicine, Xiamen University, Xiamen, Fujian, China; ^5^ Department of Clinical Laboratory, The First Affiliated Hospital of Bengbu Medical University, Bengbu, China; ^6^ Origin Quantum Computing Technology (Hefei) Co., Ltd., Hefei, China; ^7^ Anhui Province Key Laboratory of Immunology in Chronic Diseases, Bengbu Medical University, Bengbu, China

**Keywords:** tumor associated macrophages, breast cancer, macrophage polarization, immunotherapy, DLG3

## Abstract

**Background:**

M2-like tumor-associated macrophages (TAMs) promote an immunosuppressive microenvironment and contribute to tumor progression and metastasis. However, their molecular characterization and prognostic value have not been fully explored in the field of breast cancer.

**Methods:**

Weighted gene co-expression network analysis (WGCNA) was used to identify modules significantly associated with M2-like TAMs. Consensus clustering analysis identified three molecular subtypes with distinct clinical features, and we explored potential differences in genomic mutations, pathway enrichment, and immune infiltration in patients between subtypes. Machine learning algorithms were used to screen key genes and construct M2-like macrophage-associated prognostic models. Comprehensive transcriptomic analysis and *in vitro* phenotyping and polarization experiments were performed on the key gene *DLG3*.

**Results:**

M2-like TAMs infiltration was strongly associated with the prognosis of BC patients, and the associated gene characterization revealed three molecular subtypes, of which C2 has the worst prognosis with high M2 macrophages, immune desert phenotype, and immunotherapeutic resistance; C1 had the best prognosis, rich in stromal and immune cell infiltration, and metabolic pathway activation; and C3 had a high level of TILs and genomic mutations, with a high degree of immunogenicity and immunotherapeutic Potential. Risk scores can effectively predict the prognosis and immunotherapy response of BC patients, in which *DLG3* is a key gene that may be involved in shaping the immunosuppressive microenvironment of breast cancer, and down-regulation of *DLG3* can inhibit M2 polarization of macrophages.

**Discussion:**

We constructed and comprehensively solved a model of M2-like TAM-related molecular subtypes and prognosis, which helps stratify and customize treatment regimens for BC patients. We also explored the role of *DLG3* in BC progression and macrophage polarization.

## Introduction

Breast cancer (BC) is the most common malignant tumor in women worldwide, and after lung cancer is the second most common cause of cancer deaths (1). Currently, the main treatment options for BC include surgery, radiotherapy, endocrine therapy, targeted therapy, and immunotherapy (2). Among them, significant progress has been made in the treatment of HER2-positive breast cancer with targeted therapies such as trastuzumab and pertuzumab (3, 4). However, triple-negative breast cancer (TNBC) has limited treatment options due to the lack of clear therapeutic targets. Although immunotherapy and PARP inhibitors, among others, have provided new therapeutic directions for TNBC (5, 6), BC treatment still faces challenges such as the development of drug resistance, therapeutic side effects, and poor therapeutic response in advanced or metastatic BC. Therefore, there is a need to find new molecular markers and therapeutic targets to stratify and customize treatment regimens for BC patients.

In the tumor microenvironment (TME) of solid tumors, macrophages are the most abundant immune component of the innate immune system (7). Macrophages present in the tumor microenvironment are known as tumor-associated macrophages (TAMs), which are usually formed by blood monocytes or tissue-resident macrophages recruited by tumor cells to the tumor site and polarized (8). TAM are highly heterogeneous and can be polarized into different subtypes, which are mainly classified into M1-type (classically activated macrophage) and M2-type (alternatively activated macrophage). M1-type usually have anti-tumor effects, while M2 type may promote tumor growth, angiogenesis, immunosuppression and metastasis (7, 9, 10). In contrast, TAMs in tumors tend to exhibit an M2-like phenotype and play a tumor-promoting role, thus a high M1/M2 TAM ratio is associated with good survival of tumor patients (11). Hypoxic conditions in the TME may promote the formation of M2-type macrophages, while immunosuppressive molecules such as programmed death ligand 1 (PD-L1) may inhibit M1-type macrophage activity (12). Recent studies have found that Sohlh2 overexpression promotes M2 macrophage polarization, while high Sohlh2 expression in M2-like macrophages promotes TNBC progression (13). Another study found that BPIFB1 promoted M2 macrophage polarization and facilitated tumor progression in HR+BRCA (14). However, most of these studies have focused on the effects and molecular mechanisms of specific molecular targets on M2 macrophage polarization and malignant progression in BC, and no study has adequately explored the potential role of M2-like macrophage infiltration patterns in the stratification of BC patients, and the association between the level of M2-like macrophage infiltration and the immune response and prognosis of BC patients remains unclear.

Discs Large MAGUK Scaffold Protein 3 (DLG3) is a gene encoding a family of membrane-associated guanylate kinase proteins that play key roles in cell polarity and tissue morphogenesis. *DLG3* is down-regulated in glioblastoma multiforme (GBM), and its overexpression induces mitotic cell cycle arrest and apoptosis, inhibiting proliferation and migration (15). However, the role of DLG3 in BC is more complex. High expression of *DLG3* is associated with decreased survival in BC patients, and its expression is positively correlated with pathological stage and decreased survival (16). Hypermethylation of the DLG3 promoter upregulates RAC1 and activates the PI3K/AKT signaling pathway, thereby promoting breast cancer progression (17). In addition, *DLG3* is associated with sensitivity to certain chemotherapeutic agents in breast and gastric cancers, suggesting its potential role in precision medicine (18, 19). However, no studies have reported the effects and molecular mechanisms of *DLG3* on macrophages in BC.

In the present study, we first utilizing the BC single cell data (20) analyzing the effect of differences in the proportion of M2 macrophages on TME in BC patients as defined by the Cibersort algorithm, we noted that LVYE1+ macrophages were significant risk factors in BC (21). The WGCNA algorithm was used to identify modules significantly associated with M2-like macrophages, and further screened for prognostically relevant M2 macrophage-associated genes for consistency clustering analysis to classify BC patients into three distinct subtypes. Next, we screened and constructed the M2 macrophage-related gene risk score (M2GRS) by various machine learning algorithms. By correlation analysis we found that *DLG3* was significantly associated with M2 macrophage infiltration. Pan-cancer and breast cancer multi-cohort analyses further revealed the immunosuppressive role of *DLG3*. These findings will help enhance our knowledge of M2-like TAMs and guide more effective BC treatment strategies.

## Methods

### Data download and processing

Clinical information of BRCA patients as well as transcriptome sequencing data (RNA-seq), DNA methylation, single nucleotide mutations (SNVs), and copy number variants (CNVs) were downloaded from The Cancer Genome Atlas (TCGA) (https://portal.gdc.cancer.gov/), and breast cancer samples with complete survival information (n= 1073) and normal tissue controls (n=158) for subsequent analysis. The mRNA expression profiles and clinical information of the GSE20685 (n=327), GSE42568 (n=104), and GSE162228 (n=109) datasets were downloaded from the Gene Expression Omnibus (GEO) database (https://www.ncbi.nlm.nih.gov/geo/) as a validation cohort. GEO queue data is de-batched by the "normalizeBetweenArrays" function. The corresponding protein expression data for the TCGA cohorts were downloaded from the UCSC Xena database (https://xena.ucsc.edu/), which was generated and processed in the RPPA core of the TCGA Proteome Characterization Center at MD Anderson Cancer Center. The pan-cancer analysis was based on RNA-seq data and corresponding clinical information for 33 cancer types in the TCGA database, and the RNA-seq data were normalized to log2(value+1) format. Also, transcriptomics data of 20 breast cancer GEO cohorts used for pathway enrichment were downloaded from the GEO database and integrated for analysis. In addition, spatial transcriptome sequencing (ST-seq) data were used to analyze the expression specificity of DLG3 in BC, and the data were processed through the standard Seurat process. The cellular composition of each spot on 10x Visium slides was assessed using the transposed convolution analysis technique, and sample sources can be found in the **Supplementary Data**.

### Data processing and analysis of single-cell RNA sequencing

We selected 24 BRCA tumor samples with corresponding transcriptome sequencing (84,155 transcriptomes after quality control and filtering) from the breast cancer single-cell cohort GSE176078. Single-cell sequencing data were analyzed using the “Seurat” software package. Quality control (QC) was performed by retaining cells with less than 15% of mitochondrial genes and genes with expression ranging from 200 to 5000 in at least three cells. We then identified highly variable genes and set the number of highly variable genes to 2000 for subsequent analysis. Clusters of units were constructed using the “FindClusters” and “FindNeighbors” functions and visualized using the “umap” method. Finally, we performed cell annotation based on classical genealogical markers. The percentage of cellular infiltration in the TCGA cohort was calculated by transposed convolution analysis using the Cibersort website (22). Differentially expressed genes (DEGs) were identified for each cluster (FindAllMarkers) and visualized by volcano plot. scRNAtoolVis was used to map the markers in each cluster (jjVolcano). The "cytoTRACE" algorithm was used to assess the cell stemness and developmental potential of the macrophage subpopulations (23). In addition, metabolic activity was quantified at single-cell resolution by the “scMetabolism” R package using the “AUcell” method and KEGG as a reference gene set (24). The M2GRS model gene set was quantified using the AddModuleScore, Singsore, AUCell, and UCell algorithms from the “irGSEA” package. Cellular interactions between high and low-risk groups were analyzed using the “cellchat” package. In addition, the CancerSEA online database was used to study the functional status of DLG3 in BC at single-cell resolution. Single-cell sequencing data from multiple BC cohorts in the TISCH2 database were used to explore the differential expression of DLG3 among different cells (http://tisch.comp-genomics.org/home/).

### Analysis of the immune landscape

The ESTIMATE algorithm was used to assess stromal and immune scores in BC patients. The immune microenvironment was evaluated using the xCell, MCPcounter, Cibersort, TIMER, EPIC, quantiseq, and IPS algorithms of the R package “IOBR” (25). Single-sample gene enrichment analysis (ssGSEA) was performed to quantify the relative infiltration of 28 immune cell types in the TME. Immunophenotype score (IPS) was used to predict patient response to anti-CTLA-4 and anti-PD-1 data downloaded from The Cancer Immunome Atlas (TCIA, https://tcia.at/) (26). The Tumor Immune Dysfunction and Exclusion (TIDE) algorithm was also used to assess patient response to immunotherapy. T-cell and B-cell receptor (TCR&BCR) abundance scores, Tumor Infiltrating Lymphocytes (TILs) scores, and interferon-gamma (IFN-γ) scores were obtained from previous literature (27). Representative images of the mapping intensity of TILs between subtypes were obtained from a previous study (28). Cancer immune cycles were analyzed by expression scores obtained from expression profiles and compared between groups (29).

### WGCNA analysis

Expression profiles of the top 10,000 genes in the TCGA-BRCA cohort, ranked by median absolute deviation (MAD), were entered into WGCNA for subsequent analysis (30). Pearson correlation coefficients were calculated between each pair of genes to obtain a similarity matrix. The β = 5 automatically calculated by the pickSoftThreshold function and the scale-free R^2^ = 0.9 automatically calculated by the softConnectivity function were set as the softthreshold parameters to ensure a scale-free topological network and generate the TOM matrix. The correlation between the gray module and the Cibersort-defined M2 macrophage score and CD8 T cells was 0.22 and -0.18, respectively, and the gray module was selected for further analysis.

### Construction of M2 macrophage-associated molecular subtypes

A total of 4863 genes associated with M2 macrophage and CD8T cell infiltration were selected from the gray module and firstly intersected with 7812 DEGs (screening criteria: |log2FC|>1&FDR<0.05) identified by the “limma” package between the tumor tissues and normal tissues, and 2984 differentially expressed M2 macrophage-related genes were obtained (31). These genes were analyzed by univariate Cox regression. Then, 109 differentially expressed M2-like macrophage-associated genes associated with survival in the univariate were entered into the “ConsensusClusterPlus” package for consistency clustering (parameters: reps =1000, pItem =0.8, pFeature =1, clusterAlg =“km”) (32), and the optimal number of clusters was evaluated by Cumulative Distribution Function (CDF) plots and consensus heatmaps with an optimal K value of 3.

### Machine learning algorithms to validate subtype grouping and identify features

Convolutional Neural Network (CNN) was utilized to validate the classification of transcriptome data (33, 34). The dataset was normalized and coded before feeding it into a CNN architecture consisting of convolutional, maximal pooling, and fully connected layers. The model was trained for 50 episodes using the cross-entropy loss function and Adam’s optimization algorithm. The efficacy of the model was evaluated by means of a confusion matrix and a series of classification metrics, including accuracy, precision, recall, and F1 score. In addition, we identified key genes of M2 macrophage-associated subtypes based on the subtype characteristics of consensus clustering by means of the least absolute shrinkage and selection operator (Lasso) and random forest (RF) algorithms.

### Construction and validation of M2 macrophage-associated prognostic model

For the 65 M2 macrophage-associated key genes identified above using Lasso and RF, M2 macrophage-associated prognostic traits were further constructed by Lasso regression analysis and stepwise multivariate Cox regression analysis. Based on the risk coefficients obtained in the multivariate Cox regression analyses and the expression profiles of BC patients, the M2 macrophage-associated gene risk score (M2GRS) was calculated for each BC patient using the following formula: M2GRS = 0.363*DLG3-0.303*FAM111A-0.192*FOXJ1-0.126*TFPI2+0.367*CCDC9B-0.445*ERRFI1-0.174*FLT3-0.172*KRTCAP3-0.24*LRRFIP2-0.667*MAP2K6 + 1.008*PTGES3 + 0.135*SLC16A2-0.419*PSME1. BC patients were categorized into two groups, low risk, and high risk, based on the median risk score. Finally, we assessed the performance of the risk model in predicting the overall survival (OS) of patients using time-dependent ROC curves to calculate the area under the curve (AUC) values at 1, 2, 3, 4, and 5 years in the validation cohort. Column plots were constructed based on clinical characteristics and risk scores to predict BC prognosis using the “rms” package. Calibration curves were generated to assess the predictive accuracy of the model. The reliability of the model was assessed using decision curve analysis (DCA). In addition, we collected an index of previously published breast cancer prognostic models and compared the performance of M2GRS with these models.

### Functional enrichment analysis

DEGs between subtypes and risk groups were identified using the “limma” package, and genome enrichment analysis (GSEA) and gene set variation analysis (GSVA) were used to explore altered biological processes between subtypes and risk groups. The reference gene set was downloaded from the Molecular Signatures Database (MSigDB) v7.4 database. Gene sets “c2.cp.kegg.v7.4.symbols.gmt” and “h.all.v2023.1.Hs.symbols” were downloaded from MSigDB as reference gene sets. Specific biological features were obtained from the gene set (from the IMvigor210CoreBioologies package) constructed by Mariathasan et al. The “PROGENy” package was used to assess 14 signaling pathway activities in patients.

### Genomic alteration analysis

Somatic mutation and CNV data were downloaded from the TCGA website. Somatic mutation and CNV (GISTIC output) data were visualized using the R “maftools” package, and the associated tumor mutation burden (TMB) and tumor heterogeneity (MATH) scores of allelic mutations were calculated. Significant CNV amplifications and deletions were identified by GISTIC 2.0. Methylation data of TCGA cohort patients were downloaded from the GDC portal. Methylation differences between subtypes were analyzed using the R package “ChAMP” (35), which examines differentially methylated CpG between subtypes by the Wilcox test. cpGs in the X and Y chromosomes were excluded from the analysis. cpGs with FDR < 0.05 were characterized as differentially methylated CpGs.

### Prediction of immunotherapeutic response and drug response

IMvigor210, a cohort of uroepithelial cancers treated with anti-PD-L1 antibodies, was used to predict patient response to immunotherapy (36). The GSE78220 cohort, a melanoma cohort treated with anti-PD-1 (pembrolizumab or nivolumab) immunotherapy (37), and cohort GSE100797, a melanoma cohort treated with adoptive T-cell therapy (ACT) (38), were also included as predictors of response to immunotherapy. The Submap model from the GenePattern website was used to predict patient efficacy to immunotherapy. The Cancer Treatment Response Portal (CTRP) (https://portals.broadinstitute.org/ctrp) and the Parallel Analysis of Relative Inhibition in and Mixtures (PRISM) database (https://depmap.org/portal/prism/) were used to obtain drug sensitivity data for DLG3, the dose-response AUC values were used as a measure of drug sensitivity.

### Molecular docking

The 3D structure (.sdf format) of Panobinostat was downloaded from the PubChem database (https://pubchem.ncbi.nlm.nih.gov/) database. The 3D structures of the screened target proteins (.pdb format) were obtained from the PDB database (https://www.rcsb.org/) database. We first removed water molecules and ligands from the key protein structure in Pymol software and converted the pdb form to pdbqt form using OpenBabel software (https://openbabel.org). Molecular docking of the large molecule receptor (DLG3 protein) and small molecule ligand (Panobinostat) was then performed by AutoDock Tools software. Finally, we used Pymol software to visualize the molecular docking results in 3D.

### Cell culture and quantitative real-time PCR

We used MDA-MB-231, SK-BR-3, and THP-1 cells (ATCC, Shanghai, China) for *in vitro* experiments.MDA-MB-231 and SK-BR-3 cells were maintained in DMEM medium (Gibco, USA) supplemented with 10% fetal bovine serum (FBS) and 1% penicillin-streptomycin.THP-1 cells were cultured in RPMI 1640 medium containing 10% FBS, and 1% penicillin-streptomycin. Small interfering RNA (siRNA) targeting DLG3 and siRNA control were purchased from Gemma Genetics (Shanghai, China). The sequences of DLG3-targeting siRNAs were as follows: GCAGUUUCCAAAGGACAAGATT (DLG3 siRNA-1) and GGGAUGAUUGAGUCUAACATT (DLG3 siRNA-2). For transient transfection, siRNAs were transfected into MDA-MB-231 and SK-BR-3 cells using transfection reagent (Lipofectamine 2000, Invitrogen, USA) for 24 h, followed by subsequent experiments. SYBR Green qPCR mix (Vazyme, China) was used to synthesize cDNAs for real-time PCR. cDNA for real-time PCR and GAPDH was standardized as an internal reference gene. Primer sequences: DLG3, F-AAGAGGTCCTTGTATGTCAGGG, R-CACCGATCTGCTCACTTTCTC; GAPDH, F-GGAGCG AGATCCCTCCAAAAT, R-GGCTGTTGTCATACTTCTCATGG.

### Proliferation and clone formation experiments

Twenty-four hours after transfection with DLG3 siRNA, MDA-MB-231 and SK-BR-3 cells were cultured in 96-well plates (3,000 cells/well). The proliferative capacity of the treated cells was determined at 4, 24, 48, and 72 hours. The 10% Cell Counting Kit-8 (CCK8) reagent (Bio-sharp, Hefei, China) was added to each plate according to the kit instructions, and the OD 450 values were analyzed by enzyme markers (BioTek, USA). Regarding colony formation experiments, 2000 cells were inoculated in cell culture plates and allowed to grow until visible colonies were formed. We then fixed the clones with paraformaldehyde for 15 min, stained the clones with 1% crystal violet for 20 min, and counted the number of clones (>50 cells).

### Transwell migration and invasion assay

Transwell migration and invasion assays were performed by transfecting MDA-MB-231 and SK-BR-3 cells with DLG3 siRNA for 24 h and culturing them in 24-well culture plates with 8 mm pore membrane inserts to measure cell migration and invasion ability. 4 × 10^4^ cells were inoculated in the upper chamber of the transwell with 200 ul of serum-free medium, and invasion experiments required pre-coating the upper chamber with matrix gel and adding 800 μl of medium containing 10% FBS to the lower chamber. After 48 hours of incubation, transmembrane migrating cells were fixed with paraformaldehyde, stained with 1% crystal violet, and counted under a light microscope (50×).

### Flow cytometry

THP-1 cells were treated with 100 ng/ml PMA (absin, China) for 24 h to induce macrophage-like differentiation, and then maintained in a medium containing PMA for 24 h to generate M0 cells. To analyze the effect of DLG3 on macrophage polarization, THP-1-derived macrophages were co-cultured with BC (MDA-MB-231) cells in a co-culture transwell system (LABSELECT, China). BC cells were placed in the upper chamber and macrophages in the lower chamber. Co-cultured macrophages were harvested after 48 hours and incubated with PE anti-Human CD206 antibody (#1840268, absin, China) for 45 minutes on ice. Macrophages were then analyzed using cytek Dxp Athena (BD Biosciences, USA) and Flowjo software (Tree Star, USA).

### Statistical analysis

Statistical analysis and plotting were performed using R software (version 4.0.2) and GraphPad software. The Log-Rank test was used to assess prognostic differences between the two groups. Wilcoxon test was used between two paired groups. The chi-square test or Fisher’s exact test was used to compare categorical variables, and the statistical significance of the cell line experiments was evaluated by t-test in GraphPad Prism 9 software. Differences were considered statistically significant at *p < 0.05, **p < 0.01, ***p < 0.001, ****p < 0.0001.

## Results

### Research flowchart

**Figure 1:**
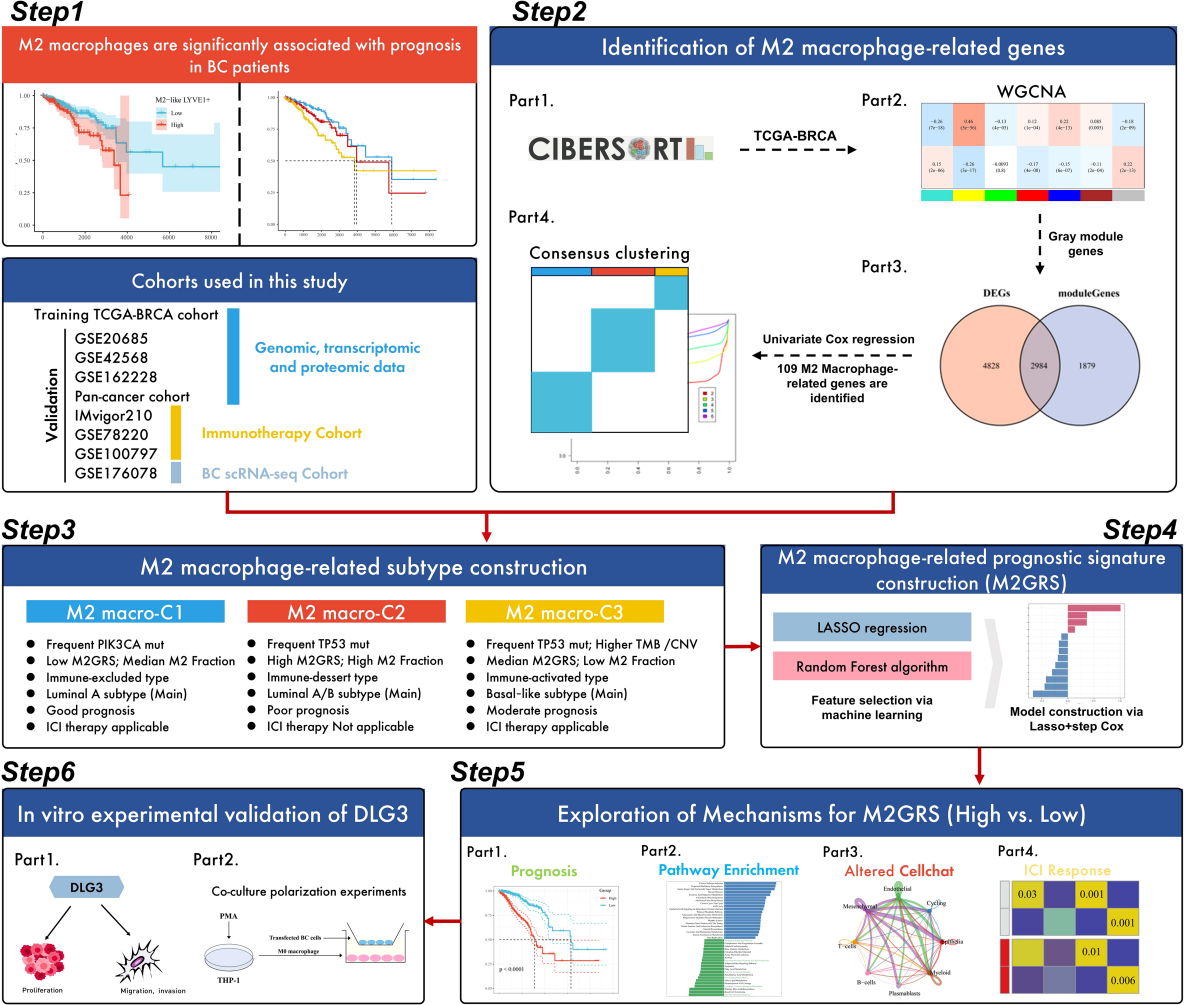
Flow chart of this study.

The workflow of this study is shown in **Figure 1**. The study design was outlined in six parts. First, we explored the prognostic value of M2-like TAMs in BC patients, i.e., high levels of M2-like TAMs have a poor prognosis. Then, M2 macrophage-related genes were identified in the second part. Specifically, the WGCNA algorithm was utilized to identify gene modules significantly associated with M2 macrophage scores calculated by Cibersort, and 109 key genes were further identified by prognostic and differential screening after extraction of the module genes, and BC was classified into 3 subtypes by consensus clustering (k-means). In the third part, we carefully analyzed the prognosis, biological processes, genomic alterations, and immune features among the subtypes, and the main features of the three subtypes are summarized in the flowchart. Next, we developed an M2-like macrophage-associated prognostic feature with a machine-learning algorithm. The feature was constructed using LASSO, RF, and multivariate Cox regression based on the TCGA-BRCA cohort. In the fifth part, we focused on exploring the potential mechanisms of risk scores in BC. To validate the prognostic value and immunotherapy prediction potential of risk profiles, we tested them on four GEO cohorts and three independent immunotherapy cohorts; potential differences in biological processes and cellular communication between high and low-risk groups were also explored. In addition, *DLG3* was found to play a key role in M2GRS and its role in the BC immunosuppressive microenvironment was explored. Finally, we performed *in vitro* experiments to investigate the role of DLG3 on the malignant progression of BC and in M2 macrophage polarization (**Figure 1**).

### Heterogeneity and prognostic value of M2-like TAM revealed by scRNA-seq

**Figure 2:**
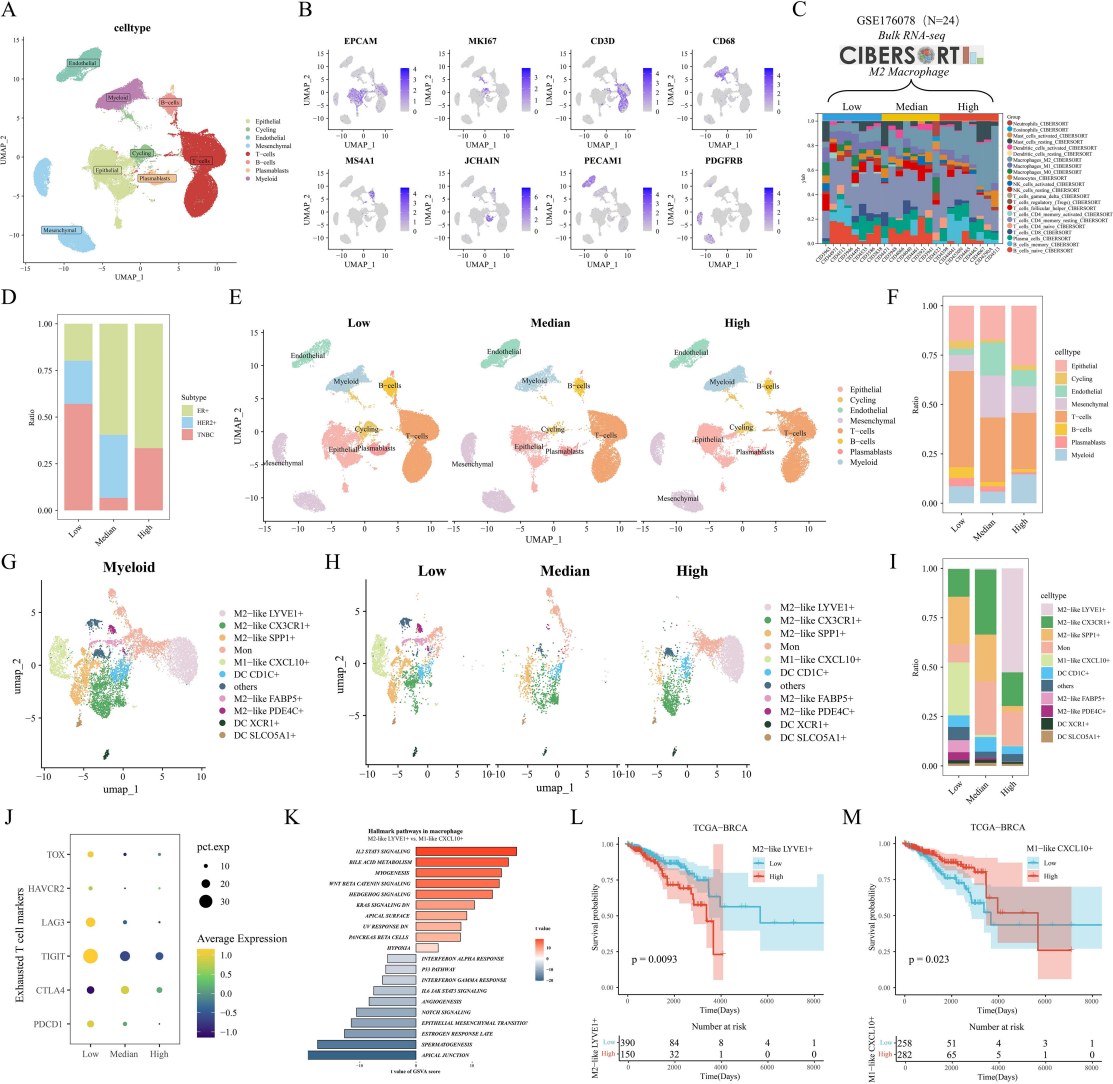
Heterogeneity and prognostic value of M2-like TAM revealed by scRNA-seq. **(A)** Single-cell UMAP downscaling of the GSE176078 cohort. **(B)** Marker gene expression distribution. **(C)** Stacked histograms grouped according to the proportion of M2 macrophages. **(D)** Differences in the distribution of clinical subtypes among the three groups. **(E)** Cellular UMAP plots grouped according to low/medium/high M2 macrophages. **(F)** Differences in immune cell grouping among the three groups. **(G)** UMAP downscaling of myeloid cell subsets. **(H)** UMAP plots of myeloid lineage grouped by M2 macrophages. **(I)** Differences in myeloid cell distribution among the three groups. **(J)** Mean expression differences of depletion markers in the three groups. **(K)** GSVA results of marker pathways between LYVE1+ macrophages and CXCL10+ macrophages. **(L, M)** Kaplan-Meier analysis of LYVE1+ macrophages and CXCL10+ macrophages in the TCGA cohort.

Our single-cell data from the GSE176078 cohort of 24 breast cancers filtered to contain a total of 84,155 cells. After descending clustering using the "umap" method, we annotated cell subpopulations and identified eight cell types, including Epithelial cells, Cycling cells, Endothelial cells, Mesenchymal cells, T-cells, B-cells, Plasmablasts, and Myeloid cells (**Figures 2A, B**). Meanwhile, breast cancer patients were further categorized into M2 macrophage Low, Median, and High groups based on the calculated M2 macrophage infiltration ratio of the 24 samples corresponding to the transcriptome sequencing results (**Figure 2C**). The bar-filled graphs showed that the Low group had a higher comparison of TNBC patients, while the High group had more ER+ patients (**Figure 2D**). Next, we observed a change from the Low group to the High group, with a significant decrease in the number of T cells and a significant increase in epithelial cells (**Figures 2E, F**). To examine the immune environment of breast cancer at high resolution, we re-clustered immune cells to identify myeloid cells, and we identified 12 clusters of myeloid cells (**Supplementary Figure S1A**), and in combination with differentially expressed genes in different clusters and classical M1/M2 marker gene expression (**Supplementary Figures S1B, C**), we annotated five M2-like (LYVE1+, CX3CR1+, SPP1+, FABP5+, and PDE4C+) macrophages, one M1-like (CXCL10+) macrophage, three dendritic cells (CD1C+, XCR1+, and SLCO5A1+), monocytes (Mon), and other cells (**Figure 2G**). Further, we found that there were more abundant FABP5+ macrophages in the Low group, while LYVE1+ macrophages were significantly enriched in the High group (**Figures 2H, I**). In addition, we assessed the differentiation potential among different cell clusters by CytoTRACE (**Supplementary Figure S1D**), and interestingly FABP5+ macrophages had the highest differentiation potential, while CXCL10+ macrophages and LYVE1+ macrophages had weaker differentiation potential. We then noted that the metabolic scores calculated by scMetabolism showed significant differences in metabolism between the three groups, with significant activation of the Arginine biosynthesis pathway in the Median and High groups, which may support the M2-type transition in macrophages (**Supplementary Figure S1E**). Pathway activity results calculated by PROGENy showed that PDE4C+ macrophages significantly activated the TGFb pathway, while LYVE1+ macrophages had the highest VEGF pathway activity (**Supplementary Figure S1F**). In addition, we noted that most T-cell exhaustion markers were highly expressed in the Low group (**Figure 2J**). GSVA enrichment results showed preferential enrichment of the WNT/β catenin pathway in LYVE1+ macrophages compared to CXCL10+ macrophages (**Figure 2K**). Finally, by the reverse convolution algorithm (CIBERSORTx) (**Supplementary Figure S1G**), we found that M2-like LYVE1+ macrophages were associated with poorer survival in the TCGA cohort, whereas M1-like CXCL10+ macrophages were a protective factor (**Figure 2L, M**).

### WGCNA identifies M2 macrophage-associated molecular subtypes

**Figure 3:**
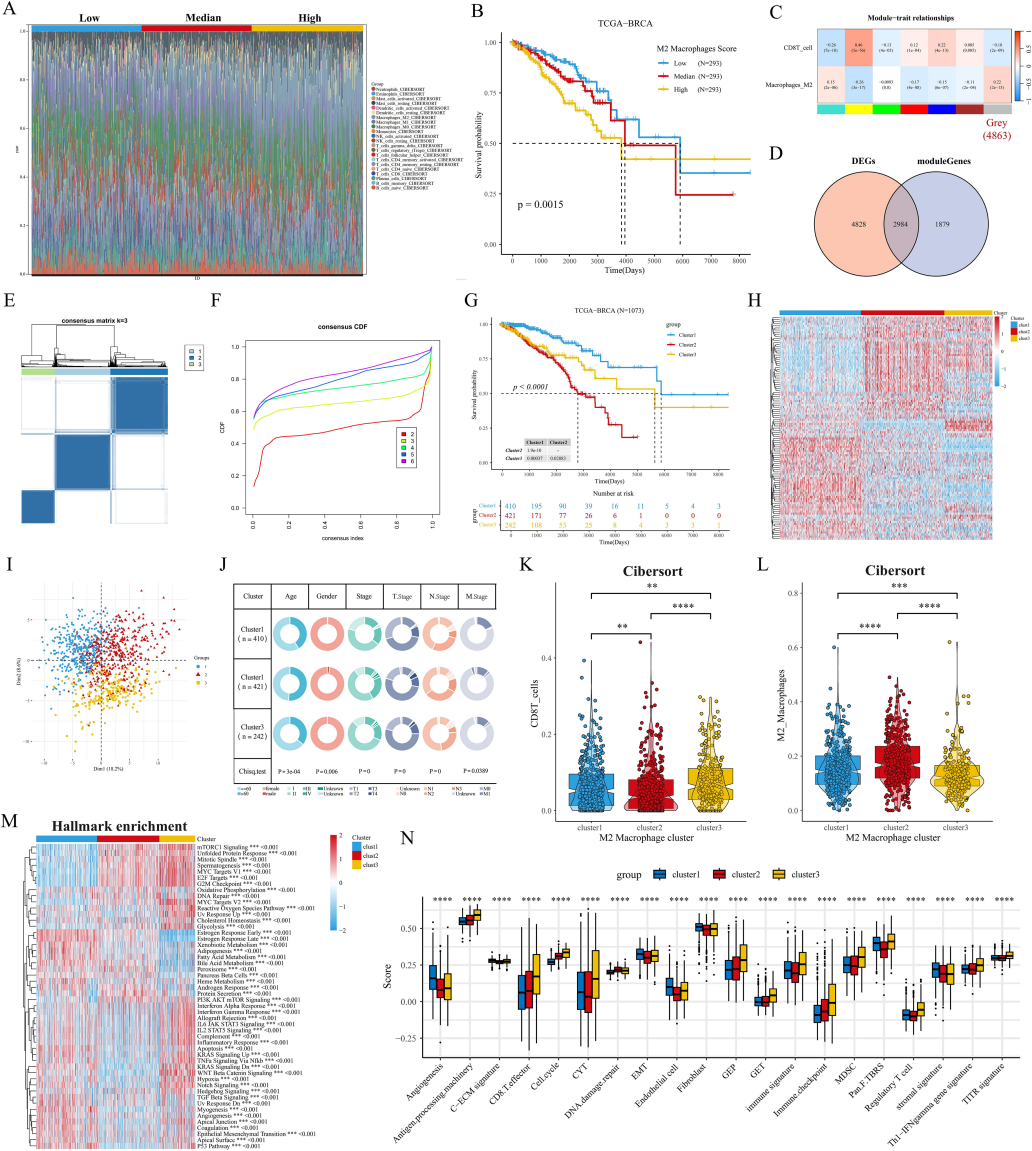
Identification of M2 macrophage-associated molecular subtypes. **(A)** Stacked histogram of the proportions of 22 immune cell types in the TCGA cohort. **(B)** Kaplan-Meier analysis shows the correlation between M2 macrophage infiltration and overall survival (OS) in the TCGA BRCA cohort. **(C)** WGCNA identifies M2 macrophage and CD8 T cell infiltration-associated modules. **(D)** Venn diagram of tumor versus normal tissue DEGs with gray modules taking the intersection. **(E, F)** Consensus clustering heatmaps and CDF curves show the 3 clusters most stable. **(G)** Kaplan-Meier survival analysis was performed to analyze the differences in OS of the three M2 macrophage-associated subtypes **(H)** Heatmap showing good separation of the three subtypes identified by the KM algorithm by features. **(I)** Sample clustering of the TCGA dataset using PCA. **(J)** Circos plots of different clinical features among the three subtypes. **(K, L)** Differences in the infiltration fraction of CD8 T cells and M2 macrophages between the three subtypes. **(M)** Heatmap showing the enriched biological pathways between the three subtypes calculated by the GSVA algorithm. **(N)** Fractional changes in specific gene sets are assessed by the GSVA algorithm. **p<0.05.***p<0.01.****p<0.001.

In the above analysis, we explored the prognostic value of macrophage subsets in breast cancer. Next, we assessed the proportion of cellular infiltration in the TCGA cohort by the Cibersort algorithm (**Figure 3A**). We categorized the patients in the TCGA cohort into Low, Median, and High groups of BRCA patients based on the proportion of M2 macrophage infiltration. After P-value screening (P<0.05), BRCA patients could be effectively prognostically stratified according to the M2 macrophage score, and M2 macrophage infiltration was significantly associated with poor prognosis (**Figure 3B**). In addition, the survival curve of the GSE20685 cohort showed similar results (**Supplementary Figure S1H**). The heatmap showed that the gray module (4863 genes) in TCGA-BRCA was negatively and positively associated with CD8 T cell and M2 macrophage infiltration, respectively (**Figure 3C**). The venn diagram showed that the gray module genes were intersected with 7812 differentially expressed genes in breast cancer to obtain a total of 2984 differentially expressed M2 macrophage-associated genes (**Figure 3D**). Further 109 M2 macrophage-associated genes with prognostic value were obtained by univariate Cox regression (P<0.05), and then we performed concordance cluster analysis. Combined with heatmap and CDF curves, we found that K=3 was the optimal clustering number (**Figures 3E, F**). Meanwhile, survival analysis showed significant prognostic differences among the three subtypes, with the C2 subtype having the worst prognosis, the C1 subtype having the best prognosis, and the C3 subtype being in the intermediate type (**Figure 3G**). In addition, we validated that in the GSE20685 cohort, the heatmap and survival curves showed that the above 109 genes could effectively stratify the prognosis of BC patients (**Supplementary Figures S1I, J**). Heatmap and PCA downscaling results showed that the 109 M2 macrophage-associated genes could effectively differentiate the three subtypes, which showed differential expression in different subtypes (**Figures 3H, I**). Clinical characterization circle plots showed that age, stage, and TNM stage showed significant differences between subtypes, with C2 having a higher proportion of terminal stage patients (**Figure 3J**). Violin plots showed that C3 had the highest level of CD8T cell infiltration; C2 had the most abundant M2 macrophage infiltration, which may be associated with its poor prognosis (**Figures 3K, L**).GSVA analysis showed that C1 significantly activated hormonal responses as well as metabolism-related pathways (e.g., adipogenesis and bile acid metabolism); relative to C1, C2 and C3 had significant activation of cell cycle- and proliferation-related pathways; notably, C3 had a higher proportion of patients with advanced stage (**Figure 3J**). activation; notably, C3 showed significant enrichment of oncogenic-related pathways (e.g., KRAS, WNT, and Notch) and immune-related pathways (e.g., interferon-gamma response, IL6 JAK STAT3) (**Figure 3M**). In addition, the heatmap of KEGG metabolism-related pathways also revealed significant metabolic differences between subtypes, especially fatty acid metabolism and tryptophan metabolism (**Supplementary Figure S1K**). To further elucidate the biological significance between the M2 macrophage subtypes of BC, we analyzed the proteomics data corresponding to the subtypes. We observed that protein expression of AR, ER, PR, and HER2 was significantly downregulated in C3, and HER2 was significantly overexpressed in C2 relative to C1 and C3; we also found that phosphorylation levels of PI3K/AKT/mTOR pathway proteins were significantly upregulated in C3 (e.g., AKT ps473 and downstream of S6 and 4EBP1, etc.), and that cell-cycle proteins B1, E1, and the kinases CDK1 were all significantly upregulated in C3 (**Supplementary Figure S1L**), and these results suggest that C3 may be associated with aggressiveness, drug resistance and poor prognosis of BC. We then performed univariate and multivariate Cox regression analyses of clinicopathologic factors and subtypes of OS. Multivariate analysis showed that both C2 and C3 were independent prognostic factors for OS in the TCGA cohort (P<0.001, HR=3.007, 95% CI=1.895-4.772; P<0.001, HR=2.879, 95% CI=1.719-4.820) (**Supplementary Figure S1M**). The ssgsea results of specific biological functions indicated that C3 appeared to have better immune infiltration (CD8T cell effect, immune profile) and immunotherapy efficacy (CYT, GEP) (**Figure 3N**). In addition, immunosuppressive cells such as MDSC and Treg were also significantly elevated in C3.

### Clinical features and genomic alterations

**Figure 4:**
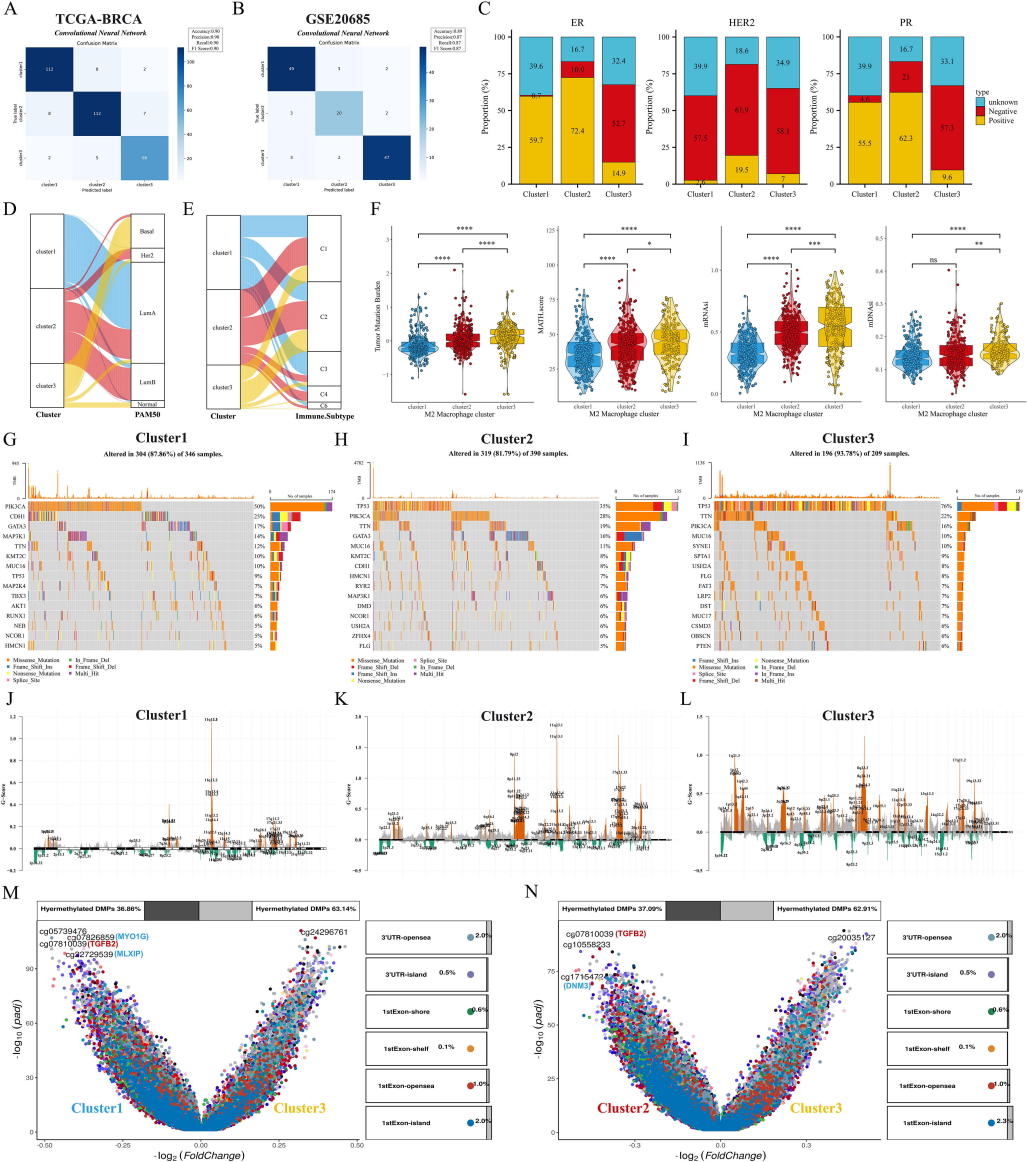
Clinical features and genomic alterations between subtypes. **(A, B)** Confusion matrix of the CNN algorithm. **(C)** Differences in the distribution of ER, HER2, and PR features among the three subtypes. **(D, E)** Sankey diagrams show the correspondence of M2 macrophage-associated subtypes with PAM subtypes and immune subtypes. **(F)** Differences in TMB, MATH, mDNAsi, and mRNAsi scores among the three subtypes. **(G–I)** Waterfall plots showing the top 15 genes in terms of mutation frequency for the three subtypes. **(J–L)** GISTIC 2.0 analysis identifies statistically significant amplifications and deletions in the three subtypes. **(M, N)** Volcano plots show changes in DNA methylation. ns: p>0.05. *p<0.05.**p<0.01.***p<0.001.****p<0.0001.

First, we verify the consistency of transcriptome classification and consensus clustering by CNN model. The performance metrics of the model: accuracy, precision, recall, and F1 score reached the 90% threshold in the TCGA cohort, while the GSE20685 cohort also had an excellent performance, which further validated the validity and reliability of transcriptome typing (**Figures 4A, B**). Bar-filled plots indicated that C1 and C2 had higher proportions of ER+, HER2+ and PR+ patients (**Figure 4C**). Sankey diagrams further showed that C3 had more patients with TNBC type, while C1 was predominantly patients with Luma A type (**Figure 4D**). In a previous study (27) immune infiltration of human tumors was classified as C1 (wound healing), C2 (INF-G dominant), C3 (inflammation), C4 (lymphocyte depletion), C5 (immune silencing), and C6 (TGF-B dominant). the C3 subtype had a higher proportion of patients with C1 and C3 types (**Figure 4E**). Next, we first analyzed the differences in TMB and MATH scores between subtypes, and patients with C2 and C3 subtypes had significant genomic instability and heterogeneity. Furthermore, the stemness index was able to describe the self-renewal capacity and differentiation potential of cancer stem cells (CSCs) in malignant tumors (39), and the results showed the same trend for the stemness index mDNAsi and mRNAsi (**Figure 4F**). To further analyze the genomic alterations among subtypes, we visualized the somatic mutation landscape among subtypes (**Figures 4G–I**). Among them, C3 had the highest overall mutation rate, C1 had frequent PIK3CA mutations, and C3 had up to 76% TP53 mutations. In addition, we utilized GISTIC2 to detect aberrant CNV regions in M2 macrophage-associated subtypes. In contrast, we observed more regions undergoing significant amplification and deletion in C2 and C3 subtypes (**Figures 4J–L**). Interestingly, the differentially methylated probe (DMP) for both C1 vs. C3 and C2 vs. C3 contained cg07810039 (TGFB2) (**Figures 4M, N**), whose down-regulation of methylation levels may affect the expression and function of the TGFB2 gene, and consequently the growth and metastasis of breast cancer cells.

### Pathway enrichment and TME landscape between subtypes

**Figure 5:**
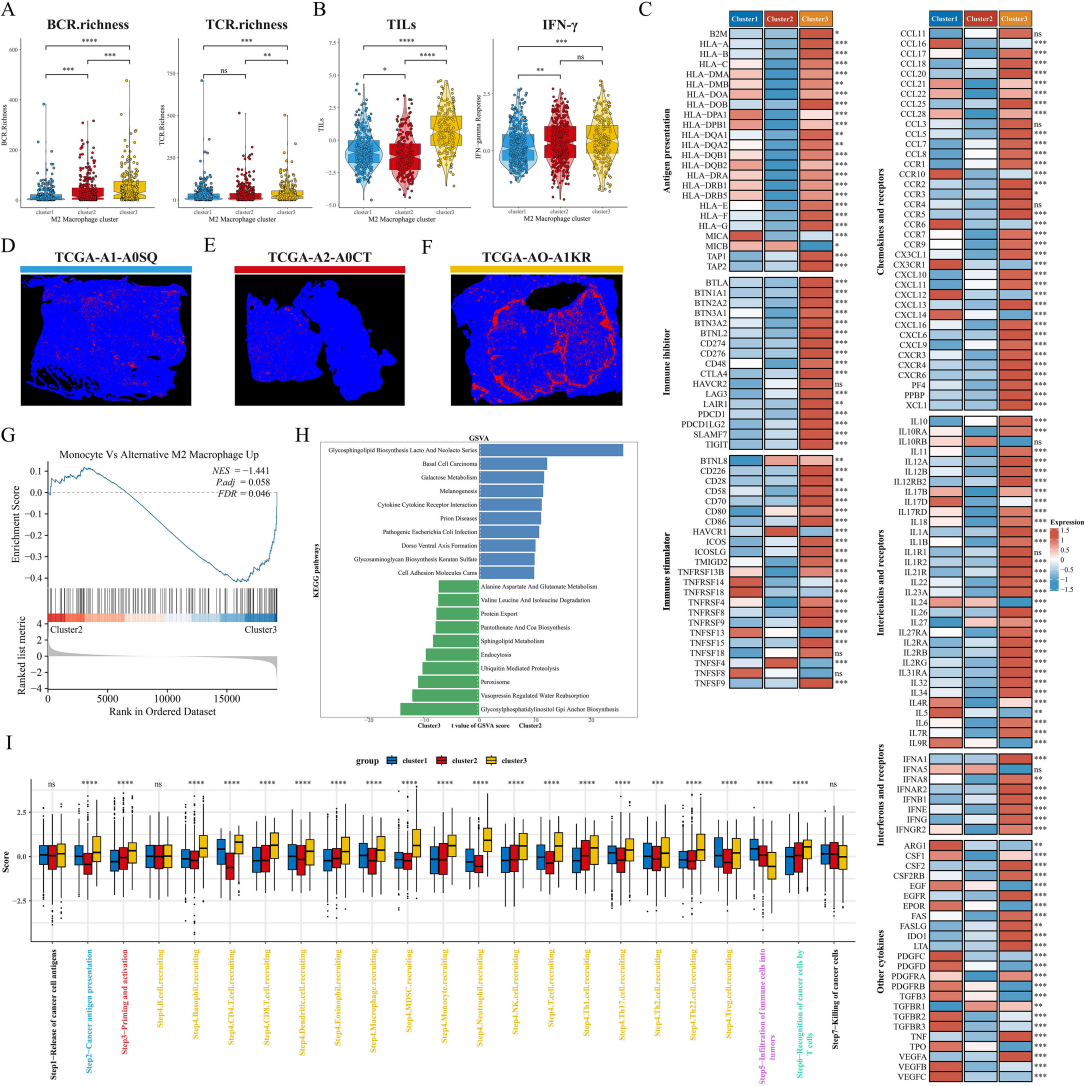
Pathway enrichment and immune landscape between subtypes. **(A)** BCR and TCR enrichment across subtypes. **(B)** Differences in TILs and IFN-γ between subtypes. **(C)** Relative expression levels of antigen presentation, immunosuppressants, immunostimulants, chemokines, interleukins, other cytokines, and their receptors in the three subtypes. **(D–F)** Mapping images of the three subtypes of TIL. **(G)** GSEA analysis shows the correlation of clusters with the M2 macrophage gene set. **(H)** The top 10 enriched KEGG pathways of C2 and C3 were explored by GSVA analysis. **(I)** Box plots showing differences in cancer immunocycle scores between the three subtypes. ns: p>0.05. *p<0.05.**p<0.01.***p<0.001.****p<0.0001.

We explored the altered pathway enrichment and TME differences among the three subtypes to further explore the potential mechanisms underlying the prognostic differences between subtypes. C1 showed the lowest diversity of BCR enrichment (all P < 0.001), while C3 showed the highest diversity of TCR enrichment, with the C1 and C2 subtypes showing no significant difference (**Figure 5A**). Meanwhile, C3 had significantly higher levels of tumor-infiltrating lymphocytes (TILs) and IFN-γ than C1 and C2 subtypes (**Figure 5B**), and previous studies have shown that high levels of TILs correlate with a favorable prognosis in TNBC and HER2 overexpressing breast cancers (40). Thus, we next analyzed the relative expression levels of antigen presentation, immunosuppressants/activators, chemokines and receptors, interferons and receptors, interleukins and receptors, and other cytokines among subtypes (**Figure 5C**). Overall, most of the genes in C2 were under-expressed, C1 appeared to be in an intermediate state, and the immune checkpoint molecules in C3 were significantly higher than those in both the C1 and C2 subtypes. In addition, we noted that the hepatitis A virus cell receptor (HAVCR1) was only highly expressed in C2, however in our previous studies we noted that HAVCR1 was also strongly associated with immune escape (41, 42), and may serve as a new target for cancer immune escape. In a previous study, Joel Saltz et al. predicted the mapping intensity of swollen TILs within the sliced area based on CNN modeling of the image blocks, and from this data we downloaded the intensity maps of TILs distribution in different subtypes of patients (**Figures 5D–F**). GSEA showed that the M2 macrophage pathway was significantly enriched in C3 (**Figure 5G**). GSVA results showed that C3 was significantly enriched in immune-related pathways (cytokine interactions, cell adhesion molecules), while C2 was closely associated with pathways such as amino acid metabolism, sphingolipid metabolism, and GPI-anchored biosynthesis (**Figure 5H**). We further explored possible alterations in the cancer-immune cycle, and we found that the activity of C3 was significantly up-regulated in the second, third, and fourth steps, but significantly down-regulated in the fifth step (immune cell infiltration into the tumor) (**Figure 5I**), which may explain the underlying mechanism of better immune infiltration and worse prognosis of the C3 subtype. Next, we quantified the level of immune cell infiltration in the BRCA cohort by means of multiple immune infiltration algorithms. First, the Estimate algorithm results showed that C1 had the highest stromal score, C3 had a significantly higher immune score than C1 and C2, and C2 had a higher tumor purity score (**Supplementary Figure S2A**). TIDE analysis showed that C1 and C3 had high TIDE scores, while C3 had a very high Merck18 gene signature (Merck18) score (**Supplementary Figure S2B**). In addition, C1 had a high MSI score and a certain level of infiltration of antitumor cells such as CD8T cells, NKT cells, and dendritic cells, and its IPS score was also at a high level, so we hypothesized that C1 tended to be an immune rejection subtype; in the heatmap, we could directly observe that C2 had a lower infiltration of all kinds of immune cells, especially reactive CD8T cells, and therefore, C2 was more consistent with the immune indifference subtype; C3 showed activation of various anti-tumor immune cells and low infiltration of M2 macrophages but also had high infiltration of suppressive Treg cells and Th2 cells, and overall C3 was closer to the immune activation subtype (**Supplementary Figures S2C, D**).

### Construction and validation of M2 macrophage-associated prognostic signatures

**Figure 6:**
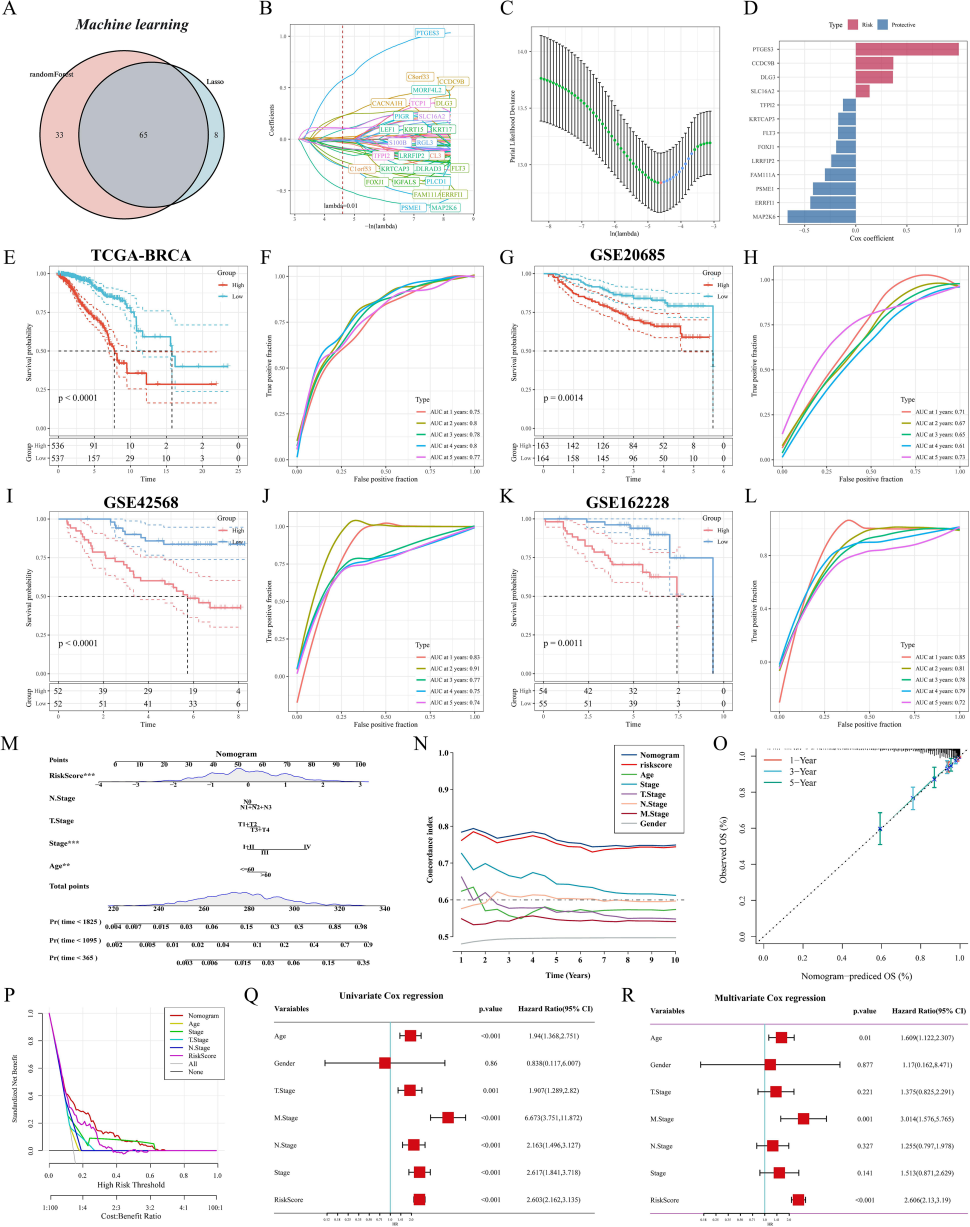
Construction and validation of M2GRS. **(A)** Venn diagram of machine learning screening of M2 macrophage-related genes. **(B)** Trajectories of each independent variable with lambda. **(C)** Coefficient distribution plots of the log(lambda) levels used for parameter selection (lambda). **(D)** Multivariate Cox coefficients for each gene in the risk model. **(E-M)** curves and ROC curves of the risk model were constructed for 13 genes in the TCGA cohort and the GEO cohort. **(M)** Column line plot combining age, staging, and risk score. **(N)** Line plots of C-index values of risk scores and clinical characteristics over time in the TCGA cohort. **(O)** Calibration curves for constructed 1-, 3-, and 5-year survival line plots. **(P)** DCA decision curve analysis. **(Q, R)** Forest plots for univariate and multivariate Cox regression analysis.

We first identified M2 macrophage-associated subtype features by lasso and RF, and the Venn diagram showed that 65 genes were considered key genes for identifying subtypes (**Figure 6A**). We further screened and constructed an M2 macrophage-associated prognostic signature consisting of 13 genes with robust predictive efficacy by lasso and stepwise multivariate Cox regression (**Figures 6B–D**). BC patients were categorized into high-risk and low-risk groups based on the median risk score. Survival analysis showed that patients in the high-risk group had shorter overall survival (OS) and time-dependent ROC curves were plotted, with area under the curve (AUC) values of 0.75, 0.8, 0.78, 0.8, and 0.77 at 1, 2, 3, 4, and 5 years, respectively (**Figures 6E, F**). Excellent predictive performance was also demonstrated in the GEO cohort, with 1- and 5-year AUCs greater than 0.7 for the GSE20685 cohort (**Figures 6G, H**) and 1- to 5-year AUC values greater than 0.7 for both the GSE42568 and GSE162228 cohorts (**Figures 6I-L**). To make M2GRS more suitable for clinical applications, we developed a nomogram by combining M2GRS with clinical features (**Figure 6M**). The line plot showed that the 1-10 years predictive C-index of the nomogram and risk score was significantly higher than that of the other clinical features (**Figure 6N**). The calibration curve showed strong agreement between nomogram predictions and actual observations (**Figure 6O**). Decision curve analysis (DCA) further showed that the nomogram exhibited robust clinical benefit relative to other clinical features (**Figure 6P**). In addition, univariate and multivariate Cox regression indicated that risk score was an independent prognostic factor (p < 0.001) (**Figure 6Q, R**).

### Model comparison and clinical characterization of M2GRS associations

**Figure 7:**
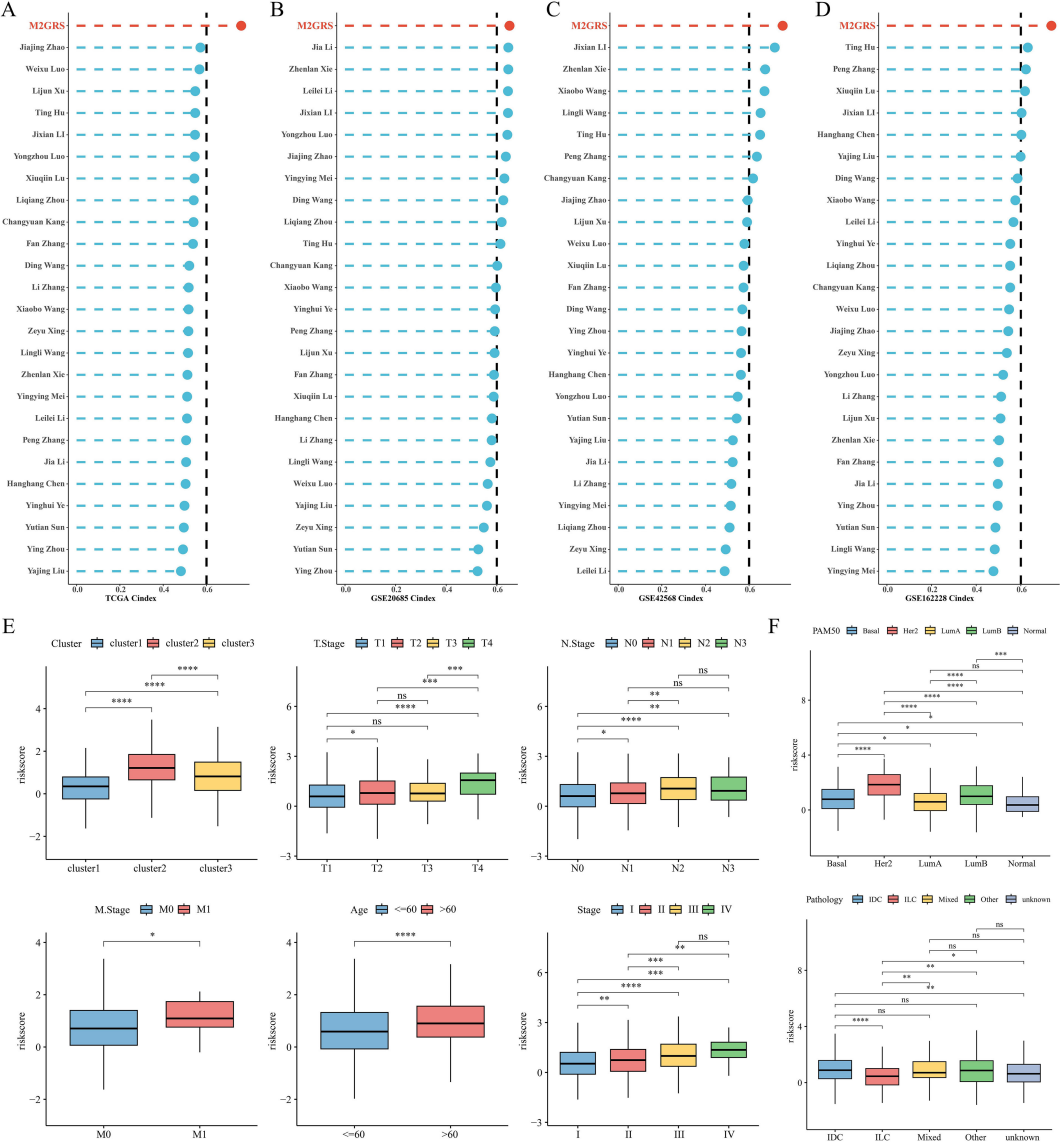
Model comparison and clinical characteristics of M2GRS. **(A–D)** Performance comparison of M2GRS with 25 published prognostic models in TCGA and three GEO cohorts. **(E, F)** Box line plots demonstrating the association between M2GRS and BC clinical features. ns: p>0.05. *p<0.05.**p<0.01.***p<0.001.****p<0.0001.

**Figure 8:**
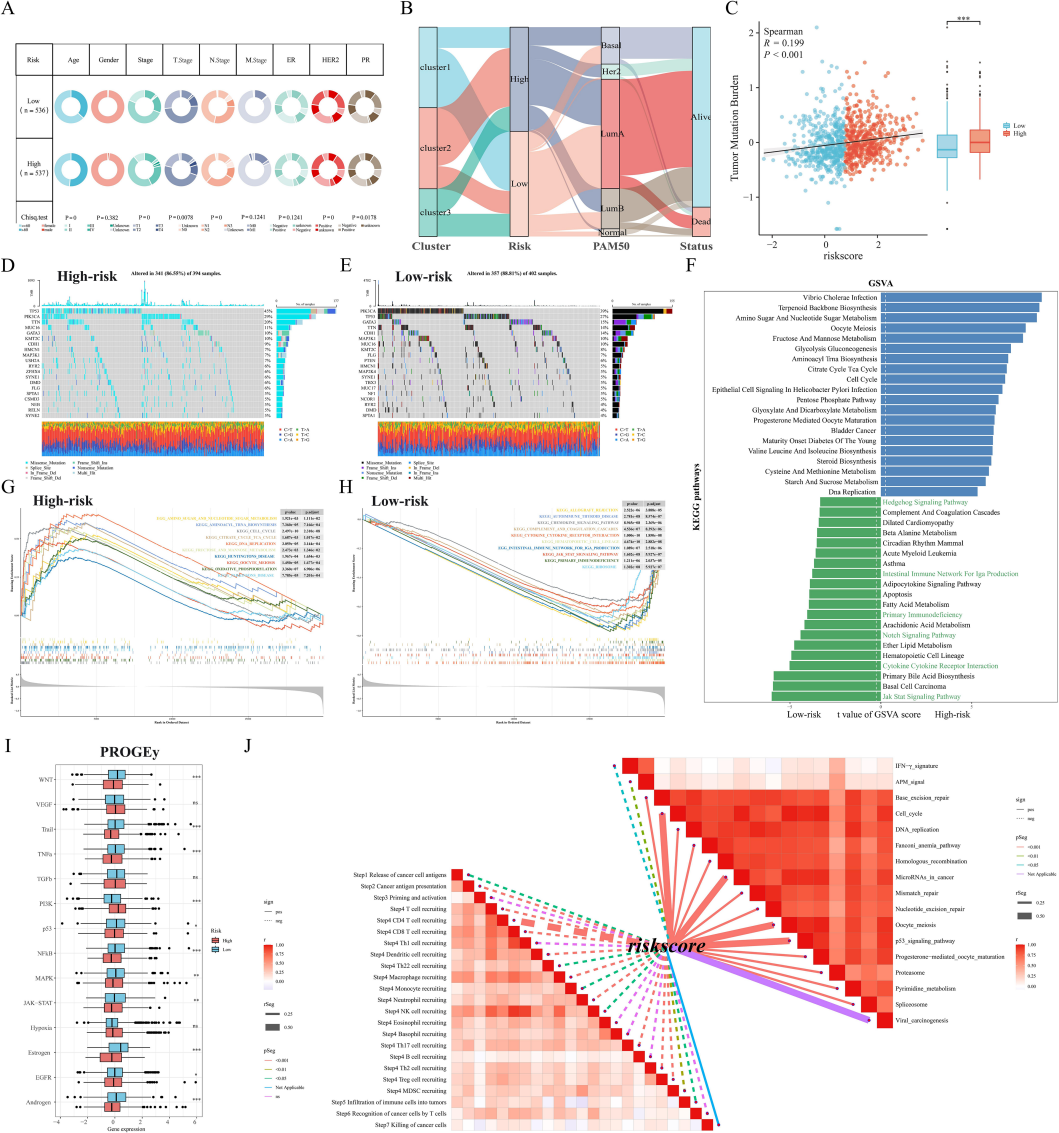
Somatic mutation and functional enrichment of M2GRS. **(A)** Correlations between M2GRS and various clinical features. **(B)** Sankey diagram demonstrating the distribution between subtypes and risk groups. **(C)** Correlation analysis between M2GRS and TMB. **(D, E)** Waterfall plots demonstrate the mutational landscapes between high- and low-risk groups. **(F)** KEGG pathway enrichment scores between high and low M2GRS groups were analyzed using GSVA and the top 20 differential pathways are shown. **(G, H)** GSEA demonstrates the enriched pathways in the high and low-risk groups. **(I)** Box line plot showing differences in 14 cancer-related pathways between high- and low-risk groups. **(J)** Correlation between M2GRS scores and steps in the cancer-immune cycle (left) and correlation between M2GRS scores and published pathway-specific feature enrichment scores (right). ns: p>0.05. *p<0.05.**p<0.01.***p<0.001.

To comprehensively compare the predictive performance of M2GRS with other prognostic features, we searched the literature on breast cancer prognostic modeling published in the last few years and included 25 different features that demonstrated that M2GRS has significantly higher C-index performance than other predictive models (**Figures 7A–D**). Next, we explored the association of M2GRS with multiple clinical features (**Figures 7E, F**). Boxplot results showed that the C2 subtype had the highest risk score in the TCGA-BRCA cohort, C3 was in the middle, and C1 had the lowest risk score; whereas the risk score increased with Stage, T.Stage, N.Stage, and M.Stage, as well as the risk score was higher in patients with Age>60 We also observed that the PAM-defined clinical subtypes Her2 type had the highest risk score, followed by LumB, and Normal type had the lowest. Invasive ductal carcinoma (IDC) and invasive lobular carcinoma (ILC) are two common histologic types, and IDC is diagnosed in approximately 75% of BC patients (43). In addition, patients with IDC had significantly higher risk scores than those with ILC and also had mixed cancers (Mixed) than those with ILC. Finally, the Clinical Characteristics circle plot showed a higher proportion of patients with Her2+ and PR+ in the high-risk group (**Figure 8A**). Finally, the Sankey diagram also showed that the high-risk group had the highest proportion of patients with the C2 subtype and that patients in the low-risk group were more likely to have the LumA type and had a better survival prognosis (**Figure 8B**).

### Somatic mutations and functional enrichment of M2GRS

First, the tumor mutation load (TMB) was significantly higher in the high-risk scoring group than in the low-risk scoring group, and correlation analysis revealed a significant positive correlation between M2GRS and TMB (**Figure 8C**). In the TCGA-BRCA cohort, changes in the distribution of somatic mutations between the low-risk and high-risk groups were investigated (**Figures 8D, E**). Patients in the low-risk group had a significantly higher frequency of somatic mutations compared to patients with high-risk scores (88.81% vs. 86.55%), especially *PIK3CA* (39% vs. 29%), *TP53* (27% vs. 45%), *GATA3* (15% vs. 10%), *TTN* (14% vs. 20%), and *CDH1* (14% vs. 9%). GSVA of KEGG pathways showed that metabolism-related pathways (e.g., aminoglycan and nucleotide glucose metabolism, glycolytic gluconeogenesis) and tumor growth pathways (e.g., cell cycle and DNA replication) were enriched in high-risk patients, whereas immunity-related pathways (JAK STAT signaling, cytokine-receptor interactions, and primary immune deficiencies, etc.) were enriched in patients with low-risk groups (**Figure 8F**). In addition, the oncogenic-related pathways Notch and Hedgehog signaling pathways were also enriched in the low-risk group. similar results were obtained by GSEA: the high-risk group was significantly enriched in metabolic and cell cycle-related pathways, and the low-risk group was mainly enriched in immune-related pathways (**Figures 8G, H**). In addition, we calculated scores for 14 cancer-related pathways and showed that the PI3K pathway was enriched in the high-risk group, whereas the low-risk group had concurrent activation of oncogenic (WNT and MAPK) and immune pathways (TNFa and JAK-STAT) (**Figure 8I**). Finally, correlation analysis of risk scores (M2GRS) revealed that risk scores were positively correlated with multiple signals such as mismatch repair, cell cycle, P53 signaling pathway, base excision repair, and viral oncogenesis, and negatively correlated with scores of IFN-γ, APM signaling, and multiple steps of the cancer immune cycle (**Figure 8J**).

### Analysis of intercellular communication between high and low M2GRS groups

**Figure 9:**
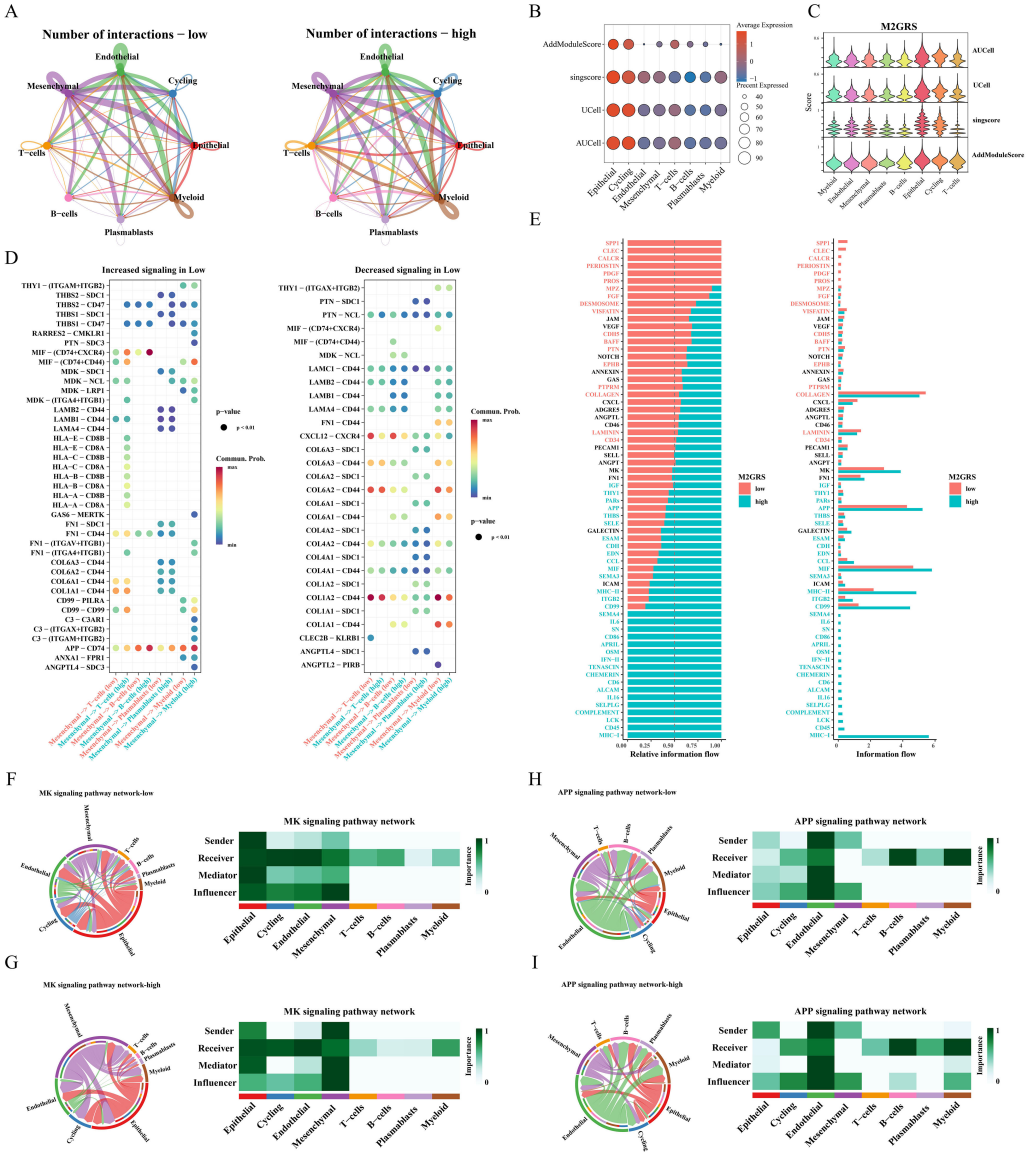
Analysis of intercellular communication between high and low M2GRS groups. **(A)** Circle plots of the number and intensity of communications between immune cells and tumor cells. **(B, C)** Bubble and violin plots showing enrichment scores for the M2GRS gene set using AUCell, UCell, singscore, and AddModulescore for each cell type. **(D)** Bubble plots of altered intercellular communication are mediated by individual signaling axes, with the horizontal axis showing the cell class that initiates and receives the signal and the vertical axis showing the receptor-ligand pairs of the signaling pathway. **(E)** Ranked bar graphs showing signaling axes of interacting networks in the high and low M2GRS groups. **(F–I)** MK and APP signaling pathways in the low and high M2GRS groups.

The cellchat algorithm was applied to explore and estimate intercellular signaling communication based on single-cell transcriptomic data from the GSE176078 cohort. The results showed that the number and interaction strength of intercellular communication increased in the high M2GRS group (**Figure 9A**). We noted that Mesenchymal cells seem to play an important role in cellular communication in BC and that the number and strength of interactions between epithelial cells (senders) and myeloid cells were increased in the high-risk group. In addition, to investigate the specific role of M2GRS in TME at the single-cell transcriptome level, we further quantified M2GRS scores at the scRNAseq level using AddModuleScore, singsore, Ucell, and AUcell algorithms. All algorithms showed higher CRS scores for epithelial and Cycling cells and lower M2GRS scores for T-cells, B-cells, and Plasmablasts (**Figures 9B, C**). The information flow of each signaling pathway was then calculated to determine the probability of communication for all cell type pairs and further quantified between Mesenchymal cells and other cells (**Figures 9D, E**). In the low M2GRS group, CD99 and FN1 pathways were reduced, MHC-I, CD45 signaling pathways were turned off, and some pathways, such as SPP1, CLEC, and PDGF signaling pathways were turned on (**Supplementary Figure S4A**). We selected two classical signaling pathways for further analysis. The communication interactions of MK and APP signaling pathways were analyzed in all cell types in the high and low M2GRS groups, in which myeloid cells both played a more important role in the high-risk group (**Figures 9F–I**).

### TME landscape and immunotherapy evaluation

**Figure 10:**
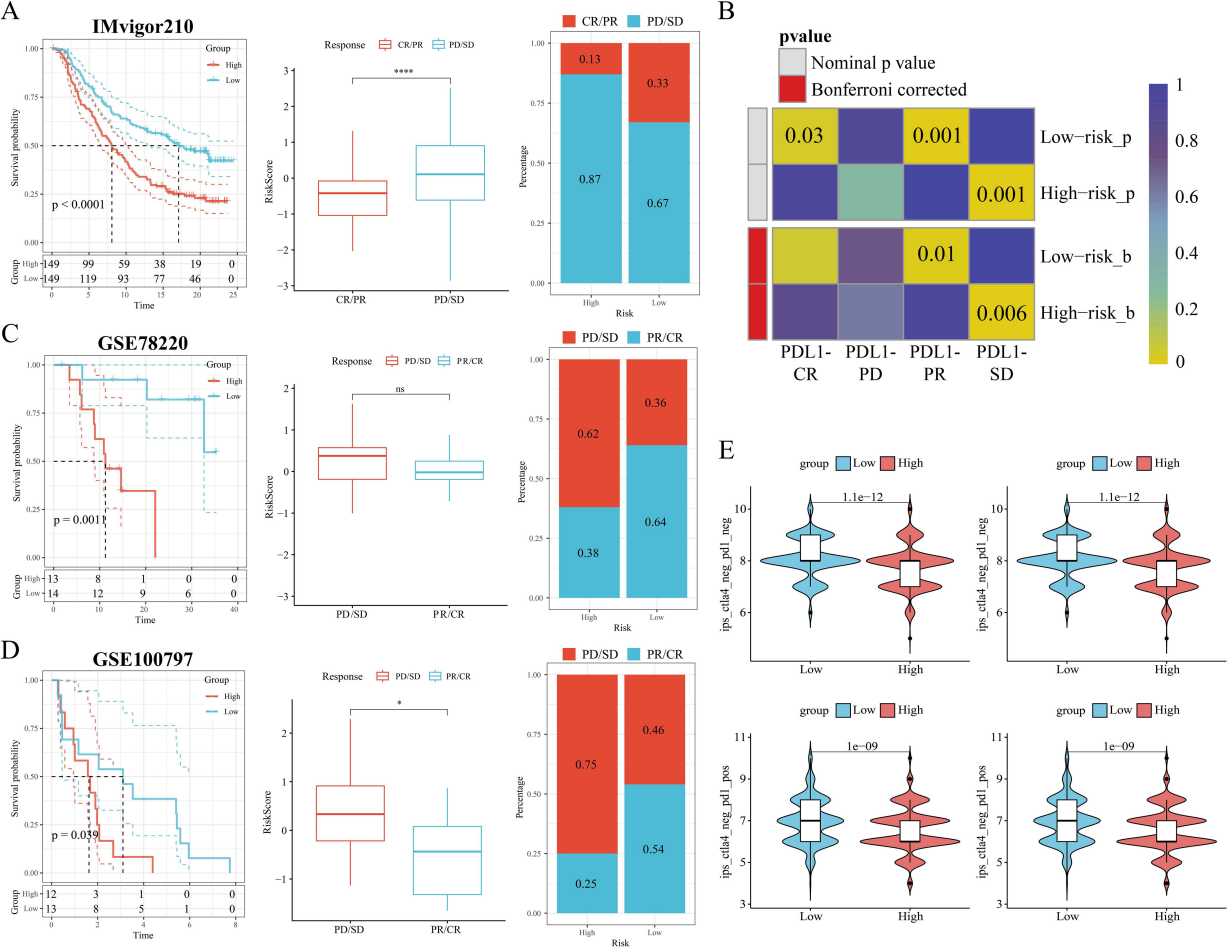
M2GRS predicts immunotherapy response. **(A)** Prognostic differences between risk-scoring groups in the IMvigor210 cohort; differences in risk scores for immunotherapy response in the IMvigor210 cohort; distribution of immunotherapy response in risk-scoring groups in the IMvigor210 cohort; **(B)** Submap analysis showing that M2GRS predicts response to anti-PD-L1 therapy. The obtained p-values were adjusted by the Bonferroni method. **(C)** Prognostic differences between risk score groups and differences in immunotherapy response scores and distribution (GSE782220). **(D)** Prognostic differences between risk score groups and differences in immunotherapy response scores and distribution (GSE100797). **(E)** Differences in IPS scores between risk groups. ns: p>0.05. *p<0.05.****p<0.0001.

Next, we further explored the TME profiles between the high and low-risk groups. First, the Estimate algorithm showed that both stromal and immune scores were higher in the low M2GRS group (**Supplementary Figure S3A**), and that either activated B cells or activated CD8T cells were significantly higher in the low M2GRS group compared to the high M2GRS group (**Supplementary Figure S3B**). The expression of antigen-presenting and immune checkpoint-associated genes was significantly down-regulated in the high M2GRS group (**Supplementary Figures S3C–E**). The infiltration fraction of M2 macrophages was significantly upregulated in the high M2GRS group, while CD8T cells were significantly downregulated (**Supplementary Figure S3F**). The heatmap also demonstrated a related trend, with lower cellular infiltration in the high M2GRS group, particularly of anti-tumor immune cells (B cells, NK cells, and CD8T cells, among others) (**Supplementary Figure S3G**). Finally, we analyzed the correlation of modeled genes with immune cells and showed that *DLG3* was simultaneously significantly positively correlated with M2 macrophages and significantly negatively correlated with CD8T cells, suggesting its important role in the BC tumor microenvironment (**Supplementary Figure S3H**). The correlation scores of M2GRS with immune cells also showed that our constructed M2GRS analysis score was significantly positively correlated with M2 macrophages and negatively correlated with CD8T cells, and dendritic cells (**Supplementary Figure S3I**). To assess the impact of M2GRS on BC immunotherapy efficacy, we selected three independently published immunotherapy cohorts for assessing the accuracy of M2GRS in predicting immunotherapy response. First, we analyzed a cohort of uroepithelial cancers treated with anti-PD-L1 therapy (IMvigor210). The low-risk group showed better prognostic outcomes compared to the high-risk group. Also, patients in the low-risk group had a higher response rate to immunotherapy (**Figure 10A**). In addition, SubMap analysis was used to assess the response to anti-PD-L1 immunotherapy in BC patients in the high-risk and low-risk groups. The results showed that the low-risk score effectively predicted partial response (PR) to anti-PD-L1 immunotherapy, while the high-risk score predicted stable disease (SD) to anti-PD-L1 immunotherapy (**Figure 10B**). In the GSE78220 cohort, we found a higher proportion of PD/SD patients in the high-risk group (**Figure 10C**). In the GSE100797 cohort, patients in the low-risk group showed good prognosis and benefited significantly from immunotherapy (**Figure 10D**). Finally, the Immunophenotype Score (IPS) was used to predict the immunotherapeutic efficacy of anti-CTLA4 and PD-1 in patients, and the violin plots showed that the scores in the low-risk group were all significantly higher than those in the high-risk group (**Figure 10E**). Overall, a prognostic signature based on M2 macrophage-related gene constructs was effective in predicting the prognosis and immunotherapy response of BC patients.

### Comprehensive analysis of the key gene DLG3

**Figure 11:**
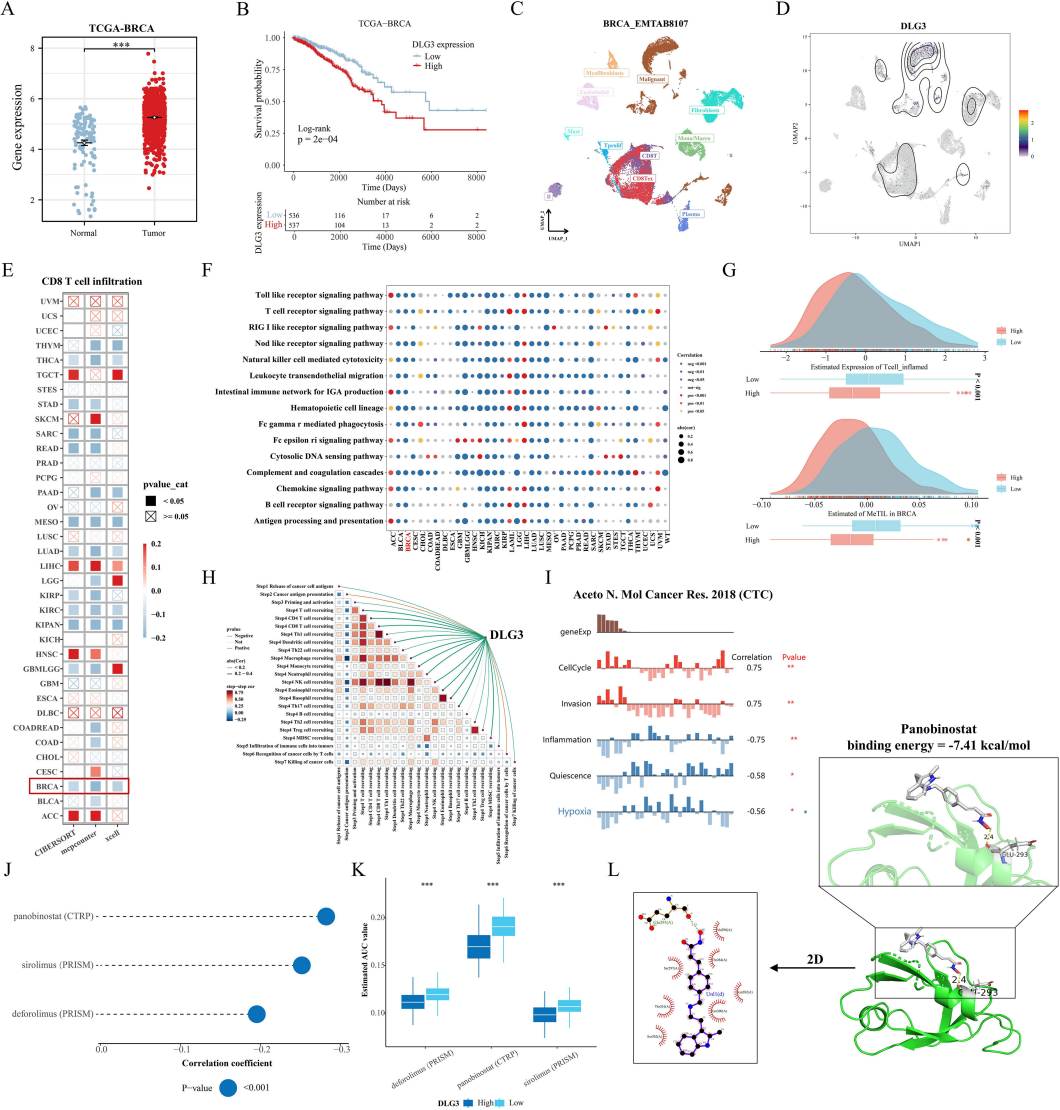
Comprehensive analysis of the key gene DLG3. **(A)** Differences in DLG3 expression in BC tumors and normal tissues. **(B)** KM survival curves grouped according to median DLG3 expression. **(C, D)** DLG3 expression in different cell types. **(E)** Correlation between DLG3 expression and CD8 T cell infiltration in the Pan-cancer cohort. **(F)** Pan-cancer GSEA analysis of immune response-related pathways between high DLG3 and low DLG3 tumor tissues. **(G)** Differences in scores between high and low DLG3 groups. **(H)** Correlation analysis between DLG3 and cancer immune circulation score. **(I)** Correlation between gene expression and different functional states in BC. **(J, K)** Results of pharmacovigilance screening based on DLG3 expression in CTRP and PRISM data. **(L)** Molecular docking of panobinostat with DLG3. *p<0.05.**p<0.01.***p<0.001.

In the above study, we found that the mRNA expression of *DLG3* was significantly positively correlated with M2 macrophages and negatively correlated with M1 macrophages and CD8 T cells, so we hypothesized that *DLG3* plays an important role in the prognostic features and may promote the formation of immunosuppressed TME in BC patients. Firstly, we analyzed the differential expression of DLG3 in the tumor tissues and normal tissues, and the results showed that *DLG3* mRNA expression in the TCGA cohort was significantly higher than that in normal tissues (**Figure 11A**). *DLG3* expression was upregulated in patients with advanced BC, with the highest *DLG3* expression level in patients with HER2 type and the lowest in Basal type (**Supplementary Figures S4B, C**). Survival analysis further showed that patients with high *DLG3* expression had a significantly lower probability of survival (**Figure 11B**). The same results were shown in the GSE20685 cohort (**Supplementary Figure S4D**). We then explored *DLG3* expression at the single-cell level, with the BRCA_EMTAB8107 cohort showing high expression of *DLG3* predominantly in malignant cells, fibroblasts, and CD8Tex (**Figures 11C, D**), and multiple BC single-cell cohorts of TISCH2 further showing that *DLG3* was expressed predominantly with subpopulations of malignant cells and cells such as Tprolif (**Supplementary Figure S4E**). To explore the inhibitory role of *DLG3* in immunomodulation, we used different algorithms to assess *DLG3* gene expression with CD8T cell infiltration in the Pan-cancer cohort. The results showed that *DLG3* expression was negatively correlated with CD8T cell infiltration in a variety of cancers (**Figure 11E**). GSEA demonstrated that many immune-related pathways, such as T-cell receptor signaling and Nod-like receptor signaling, were enriched in patients with high *DLG3* expression in most cancer types (**Figure 11F**). In addition, GSVA showed that *DLG3* high expression was significantly enriched for metabolic processes such as GPI-anchored biosynthesis, biotin synthesis, and others (**Supplementary Figure S4F**). Previous studies have shown that tumors with high inflammatory T-cell scores typically have more T-cell infiltration, which may correlate with better immunotherapy outcomes (44). In addition, the MeTIL score is a score calculated based on DNA methylation and is used to assess tumor-infiltrating lymphocytes (TILs), especially CD8+ TILs (45), high levels of TILs contribute to increased responsiveness to neoadjuvant chemotherapy and survival in BC patients, especially in HER2-positive and TNBC breast cancer patients (40). **Figure 11G** shows that patients with low *DLG3* expression have higher T cell inflamed scores and MeTIL scores, which may represent a better immunotherapy efficacy and survival prognosis with low *DLG3*. In addition, analysis of multiple immunotherapy cohorts also showed the predictive potential of *DLG3* for immunotherapy (**Supplementary Figure S4G**). Correlation analysis demonstrated that *DLG3* was negatively associated with multiple steps of the cancer immune cycle, potentially influencing anti-tumor immune processes and promoting the formation of an immunosuppressive microenvironment (**Figure 11H**). To capture gene expression at single-cell resolution and its correlation with the functional status of cancer in BC, the dataset used RNA-seq data from circulating tumor cells (CTC) in the blood of metastatic ER-positive BC, as shown in Figure, *DLG3* expression was positively correlated with the cell cycle and invasive functional status, and negatively correlated with inflammatory status in BC (**Figure 11I**). Finally, to explore clinical therapeutic options with *DLG3* as a potential target, we predicted the differences in drug sensitivity between high and low *DLG3* groups using CTRP and PRISM databases, and the results showed that three groups of drugs were significantly negatively correlated with *DLG3* expression, including: panobinostat (HDAC inhibitor, targeted drug), sirolimus (mTOR inhibitor, immunosuppressant) and deforolimus (mTOR inhibitor, targeted drug) (**Figures 11J, K**). The 2D and 3D structures of panobinostat were obtained from the PubChem database, and panobinostat was molecularly docked to DLG3. The binding energy of the docking is usually considered to be <-5 kcal/mol, and there is a good binding capacity between the two. Using AutoDock Tools software, the docking results showed good binding between panobinostat and DLG3, and DLG3 may be a potential binding target for panobinostat (**Figure 11L**).

### DLG3 shapes the immunosuppressive microenvironment of BC

**Figure 12:**
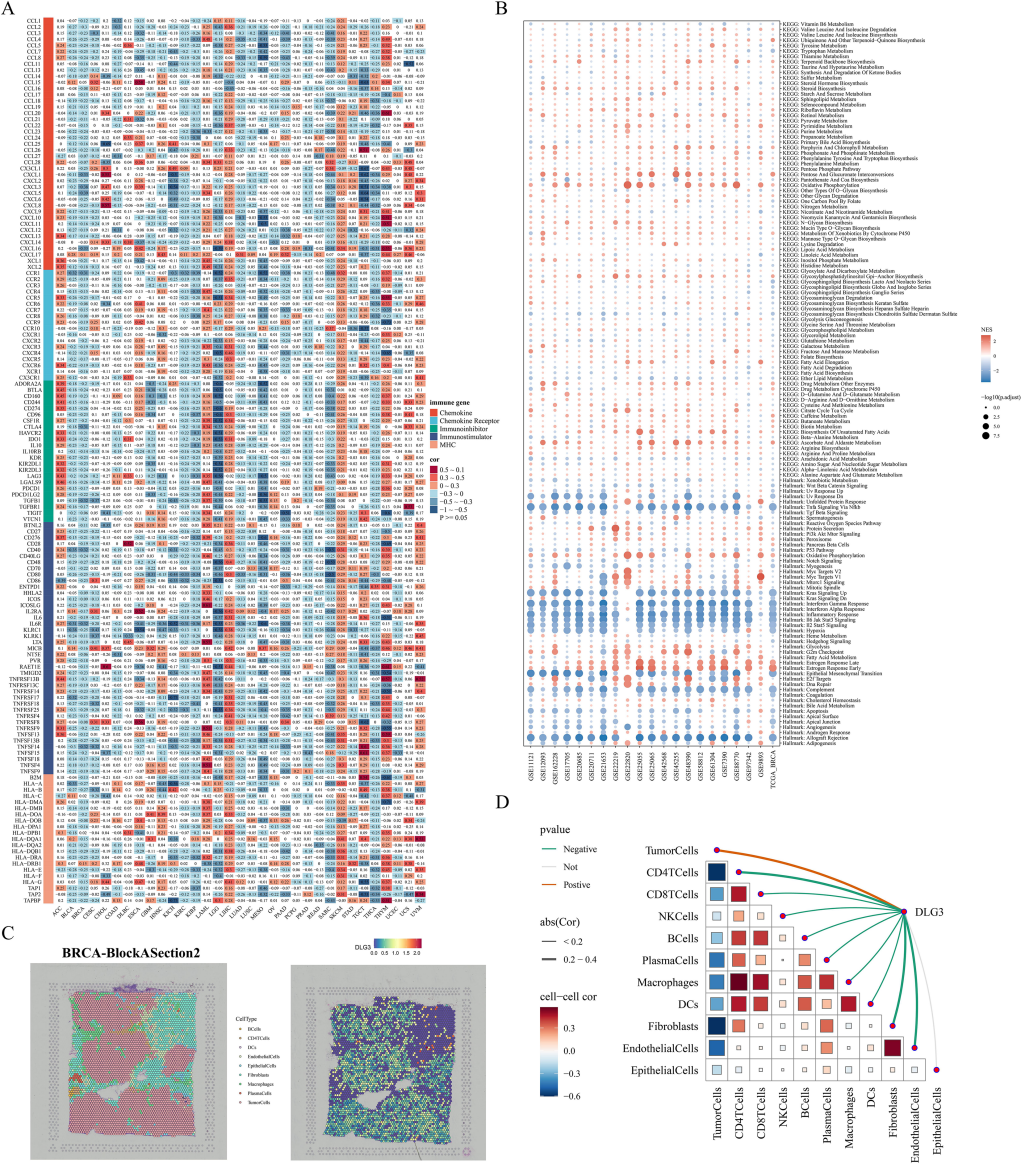
DLG3 shapes the immunosuppressive microenvironment of BC. **(A)** Heatmap of the correlation between DLG3 and immune-related genes in a pan-cancer cohort. **(B)** Bubble map of GSEA enrichment in TCGA and 20 BC cohorts. **(C)** Cellular localization and expression of the spatial transcriptome. **(D)** Spearman correlation analysis of cell-to-cell, and DLG3 expression to cell.

In the above analysis, we noted that *DLG3* may affect immunotherapy efficacy in BC patients and is mainly highly expressed in malignant or suppressive cell subpopulations, so we explored the potential role of *DLG3* in the immunosuppressive microenvironment of BC in a pan-cancer cohort and multiple cohorts of breast cancer. First, correlation heatmaps showed that *DLG3* expression was significantly negatively correlated with the vast majority of MHC molecules, cytokines and their receptors, and immunosuppressor/activator genes in BRCA, in addition to a similar trend in cancers such as BLCA, KIRC, etc. (**Figure 12A**). GSEA of the TCGA cohort and the 20 GEO public cohorts demonstrated that *DLG3* was associated with interferon α/γ response, inflammatory response, TNFα signaling, IL6 JAK STAT3 signaling, and IL2 STAT5 signaling, etc., and was significantly positively correlated with the estrogen response pathway (**Figure 12B**). The above results suggest that *DLG3* may be involved in the development of BC and the formation of the immunosuppressive microenvironment. Spatial transcriptome results of multiple BRCA samples all showed that *DLG3* was mainly localized in tumor cells, while partially expressed in epithelial cells, macrophages, and fibroblasts (**Supplementary Figure S4H**). **Figure 12C** is a spatial transcriptome deconvolution that was utilized to show cellular composition and maxima at each point to provide a spatial map of the cellular distribution within the tumor. map, the results showed that *DLG3* was highly expressed mainly in the tumor cell region. Spearman correlation analysis showed that *DLG3* was negatively correlated with all kinds of immune cells and positively correlated with tumor cells (**Figure 12D**). In addition, the histogram showed that *DLG3* was mainly expressed in malignant areas (**Supplementary Figure S4I**).

### Role of DLG3 in BC progression and macrophage polarization

**Figure 13:**
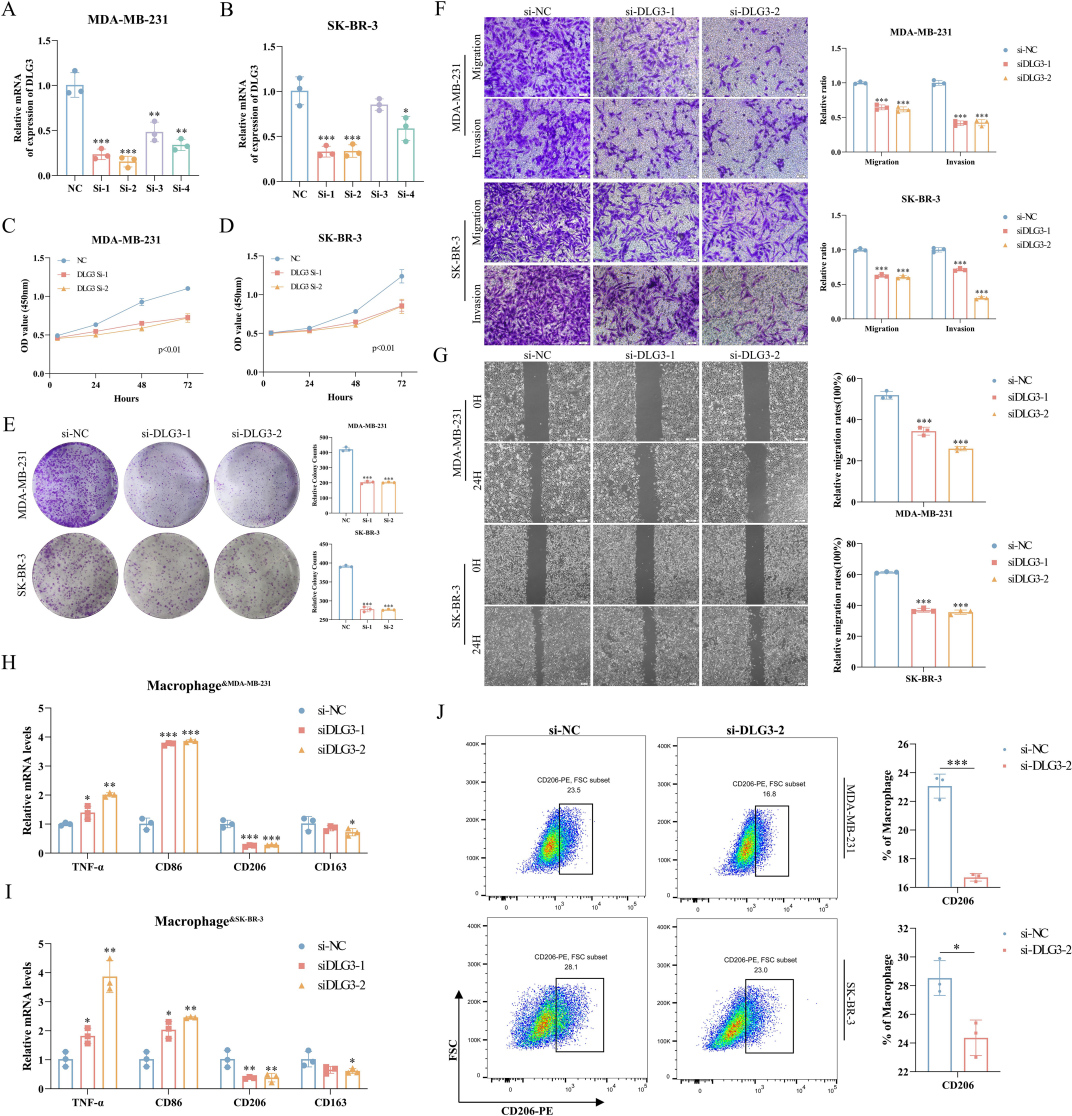
Role of DLG3 in BC progression and macrophage polarization. **(A, B)** RT-qPCR to verify the interference efficiency of DLG3. **(C, D)** CCK8 assay for knockdown of DLG3. **(E)** Knockdown of DLG3 in clonal speckle formation assay. **(F, G)** Transwell and wound healing assays of knockdown DLG3. **(H, I)** Relative mRNA expression of TNF-α, CD86, CD206, and CD163 in THP-1-derived macrophages was detected by RT-qPCR after co-culture with control or DLG3 knockdown BC cells. **(J)** Co-culture treatment of THP-1 cells followed by analysis of THP-1 polarization by flow cytometry. *p<0.05.**p<0.01.***p<0.001.

We first assessed the efficiency of specific siRNA silencing of *DLG3* expression in two cell lines, MDA-MB-231 and SK-BR-3, and the interferences with higher knockdown efficiencies were selected for subsequent experiments (**Figures 13A, B**). CCK8 assay showed that knockdown of *DLG3* significantly inhibited the proliferative ability of BC cells (**Figures 13C, D**. Clonogenic speckle formation assay showed the same trend (**Figure 13E**). The results of Transwell assay showed that knockdown of *DLG3* significantly inhibited the migration and invasion ability of BC cells (**Figure 13F**). In addition, wound healing assay also showed that knockdown of *DLG3* significantly inhibited the migration ability of BC cells (**Figure 13G**). We then co-cultured control and *DLG3* knockdown BC cells with PMA-induced THP-1 macrophages in a Transwell system (**Figure 1** Step 6). RT-qPCR results showed that *DLG3* knockdown BC cells enhanced the expression levels of M1 biomarkers (TNF-α and CD86) and down-regulated the M2 biomarker expression (CD206 and CD163) (**Figures 13H, I**). Flow cytometry analysis further showed that the proportion of M2 macrophages (CD206 as a marker) was significantly reduced after co-culture with *DLG3* knockdown BC cells (**Figure 13J**). This suggests that *DLG3* plays an important role in promoting macrophage polarization to the M2 phenotype.

## Discussion

Due to the heterogeneity and prognostic differences of BC, powerful classifiers are of great research value for precision medicine and improvement of prognosis, and the study of its molecular subtyping has been the focus of bioinformatics. The research results by Perou et al. are epoch-making for molecular typing of BC, which classified BC into four major molecular subtypes, LuminalA, Luminal B, HER-2 overexpression, and Basal-like type, through clustering analysis of gene expression profiles (46). Different gene expression patterns reflect fundamental differences in tumor cell biology and, more importantly, correlate significantly with clinical outcomes, and BC intrinsic typing has been widely recognized and applied (47). A study by Jiang et al. provided an in-depth analysis of 465 cases of triple-negative breast cancer (TNBC) by genomic and transcriptomic sequencing, and classified TNBC into four subtypes: luminal androgen receptor (LAR), immunomodulatory (IM), basal-like immune- suppressed (BLIS) and mesenchymal-like (MES). This typology not only reveals the complexity of TNBC at the molecular level, but also shows significant differences in prognosis and response to therapy between the different subtypes (48). The above studies suggest that molecular subtyping studies of BC are important for understanding the heterogeneity of BC, guiding individualized treatment, and improving patient prognosis.

High infiltrating abundance of TAM is associated with poor prognosis in a variety of tumors, and most TAM are expressed in the M2 phenotype (12). M2 macrophage infiltration has been previously documented for melanoma (49) and gastric cancer (50) squamous cell carcinoma of the head and neck (51) and other cancers with predictive value for prognosis and immunotherapy, but there is no study in breast cancer to synthesize the clinical value of M2-like macrophage pairing with breast cancer patients by bioinformatics, in this study, we first explored the difference in immune cell composition of the three levels of M2 macrophage infiltration of Low/Median/High in the single-cell transcriptome, in which we noted that in the High M2 macrophage group had the highest percentage of LYVE1+ macrophages, and survival curves by back-convolution to the TCGA cohort also indicated that their high expression had poorer OS and significantly activated the Wnt/β catenin signaling pathway. Previous studies have confirmed that activated Wnt/β-catenin signaling promotes M2 macrophage polarization (52), and that LYVE1+ macrophages express typical M2 macrophage markers (CD163, CD206) and exert a wide range of homeostatic and tissue repair functions (53). LYVE-1+ macrophages in the BC tumor microenvironment may affect tumor progression and immune responses through multiple mechanisms, such as forming multicellular nesting structures, inducing immunosuppression, and influencing tumor angiogenesis and metastasis (21). Next, we identified three different subtypes based on M2 macrophages using TCGA-BRCA as a training set, which were validated in the GEO cohort. By comprehensive multi-omics analysis among subtypes, we concluded that C1 had the best prognosis, moderate M2 macrophage infiltration, metabolically active, high stromal and immune infiltration, and predominantly Luminal A subtype; C2 had the worst prognosis, had the highest M2 macrophage infiltration and M2GRS, had a moderate level of genomic mutations, and low expression of immune-related genes, and was biased toward and immune-deserted type. In contrast, C3 has an intermediate prognosis, high levels of genomic mutations, abundant immune infiltration, predominantly TNBC pathologic subtype, and immunotherapy sensitivity. Combined with proteomic data of the subtypes, targeting these specific biological features may require the development of novel therapeutic strategies, such as inhibitors against the PI3K/AKT/mTOR pathway, and targeted therapies against HER2, to enhance therapeutic efficacy and improve patient prognosis. In addition, immunotherapy is playing an increasingly important role in the treatment of TNBC, which is considered a potential candidate for immunotherapy due to its high PD-L1 expression, high TMB, and more TILs (6, 54, 55), in metastatic TNBC (mTNBC), immunotherapy in combination with chemotherapy (e.g., nab-paclitaxel) has shown progression-free survival (PFS) and OS benefits in selected populations (PD-L1-positive subgroups) (56). New immunotherapy strategies are being explored, including the combination of CXCR2 inhibitors with ICIs (57), and the use of nanotechnology to develop PmTriTNE@CDA, a CD44×PD-L1/CD3 tri-specific T-cell nanoadapter for enhancing TNBC immunogenicity (58).

In order to facilitate clinical application and quantify the M2 macrophage score, we further identified the core genes for subtype identification by machine learning algorithms (RF and Lasso) based on the above key transcriptomic features for typing, and constructed a robust and reliable M2 macrophage-associated prognostic model with strong predictive ability by multivariate Cox. Not only its AUC is greater than 0.7 in both TCGA training set and GEO validation set, but also compared with the breast cancer-related prognostic models published in recent years, our M2GRS exhibits a significant performance advantage, and we preliminarily demonstrated that M2GRS can be used to predict the response to immunotherapy through the immunotherapy cohort. In addition, in the pathway enrichment between high and low risk groups, we noticed that the high-risk group was significantly enriched in metabolic pathways such as amino acid and nucleotide metabolism, glycolysis, and the citric acid cycle, while the relatively low risk group activated both oncogenic and immune response pathways. Moreover, M2GRS was significantly negatively correlated with multiple steps of the cancer immune cycle and significantly positively correlated with M2 macrophage infiltration, suggesting that our construct M2GRS correlates with a variety of biological features of BC with strong predictive power. In addition, previous studies suggested that our MK and APP pathways could promote BC angiogenesis and immune escape (59), we noticed that myeloid cells with high M2GRS played a more important cellular communication role in the MK and APP pathways, which might represent a stronger angiogenic and immune escape potential in the high M2GRS group. Then, we identified a specific role of *DLG3* by correlation analysis, whose role in TME has not been revealed. Based on the comprehensive analysis of bulk transcriptome, single-cell transcriptome and spatial transcriptome, we noticed that *DLG3* was not only negatively correlated with CD8T cells and positively correlated with M2 macrophages, but also associated with several indicators of immunotherapy (inflammatory T cells, cancer immune cycle, etc.), and the subsequent multi-cohort analysis confirmed that *DLG3* had some predictive potential for immunotherapy. And the correlation analysis with immunosuppressants, immune activators, MHC molecules and cytokines and other immune-related genes, and the GSEA enrichment analysis of 21 transcriptomics cohorts of breast cancer further confirmed that *DLG3* might be involved in shaping the immunosuppressive microenvironment of BC. Macrophages are highly plastic and can be induced into the M2 phenotype by tumor cells. We speculated whether high expression of DLG3 in BC cells would induce M2 polarization of macrophages in BC TME, and our subsequent *in vitro* experiments confirmed this conjecture.

However, our study still has some limitations. First, this study primarily analyzed public databases, so inherent case selection bias may have influenced the results, and further validation in larger prospective trials is needed to assess the value of clinical applicability of M2 macrophage-associated molecular subtypes and prognostic models. Second, we investigated differences in response to M2GRS-predictive immunotherapy using cohorts such as IMvigor210 and the applicability to BC patients remains to be further validated in clinical trials. Third, more combined histological information is needed for comprehensive analysis to fully resolve macrophage dynamics in BC and for precise quantification. Finally, the key gene *DLG3* requires further *in vivo* experiments to validate its functional role in BC and uncover potential molecular mechanisms.

Overall, our study identified a novel and reliable M2-like TAM-associated molecular subtype and constructed a prognostic model, which can be used to predict OS and immunotherapeutic response in BC, and also explored the potential role of *DLG3* in the immunosuppressive microenvironment of BC. Our study was able to broaden the understanding of the role of M2-like TAMs in BC biology and prognostic prediction, and *DLG3* is expected to be a novel predictive biomarker of BC prognosis and immunotherapeutic response.

## Data Availability

The original contributions presented in the study are included in the article/**Supplementary Material**. Further inquiries can be directed to the corresponding authors.
