# Supplementary material for: Molecular characterization and prognostic modeling associated with M2-like tumor-associated macrophages in breast cancer: revealing the immunosuppressive role of DLG3
Source: Front Immunol. 2025 Aug 13;16:1650726. doi: 10.3389/fimmu.2025.1650726 (PMC12380917; doi:10.3389/fimmu.2025.1650726)
Supplement: Supplementary file 1 [file DataSheet1.docx]

**Supplementary Figure**





**Supplementary Figure S1.** (A) Myeloid UMAP plot. (B) Dot plot of M2 macrophage-associated marker expression. (C) Volcano plots demonstrating differentially expressed genes in each cell cluster. (D) The cytoTRACE algorithm calculates tSNE results for each myeloid cell cluster. (E) Metabolic differences among the three groups of myeloid cells. (F) Pathway activity differences in myeloid cells. (G) Heatmap of Cibersort's deconvolution results. (H) Differences in survival prognosis between high and low M2 macrophage subgroups in the GSE20685 cohort. (I) Heatmap of consensus clustering for the GSE20685 cohort. (J) Survival analysis of M2 macrophage-related subtypes in the GSE20685 cohort. (K) Differences in metabolic pathway activation among the three isoforms of the TCGA cohort. (L) Heatmap showing pathway protein expression levels corresponding to the three subtypes of the TCGA cohort. (M) Univariate and multivariate Cox regression forest plots of the TCGA cohort.


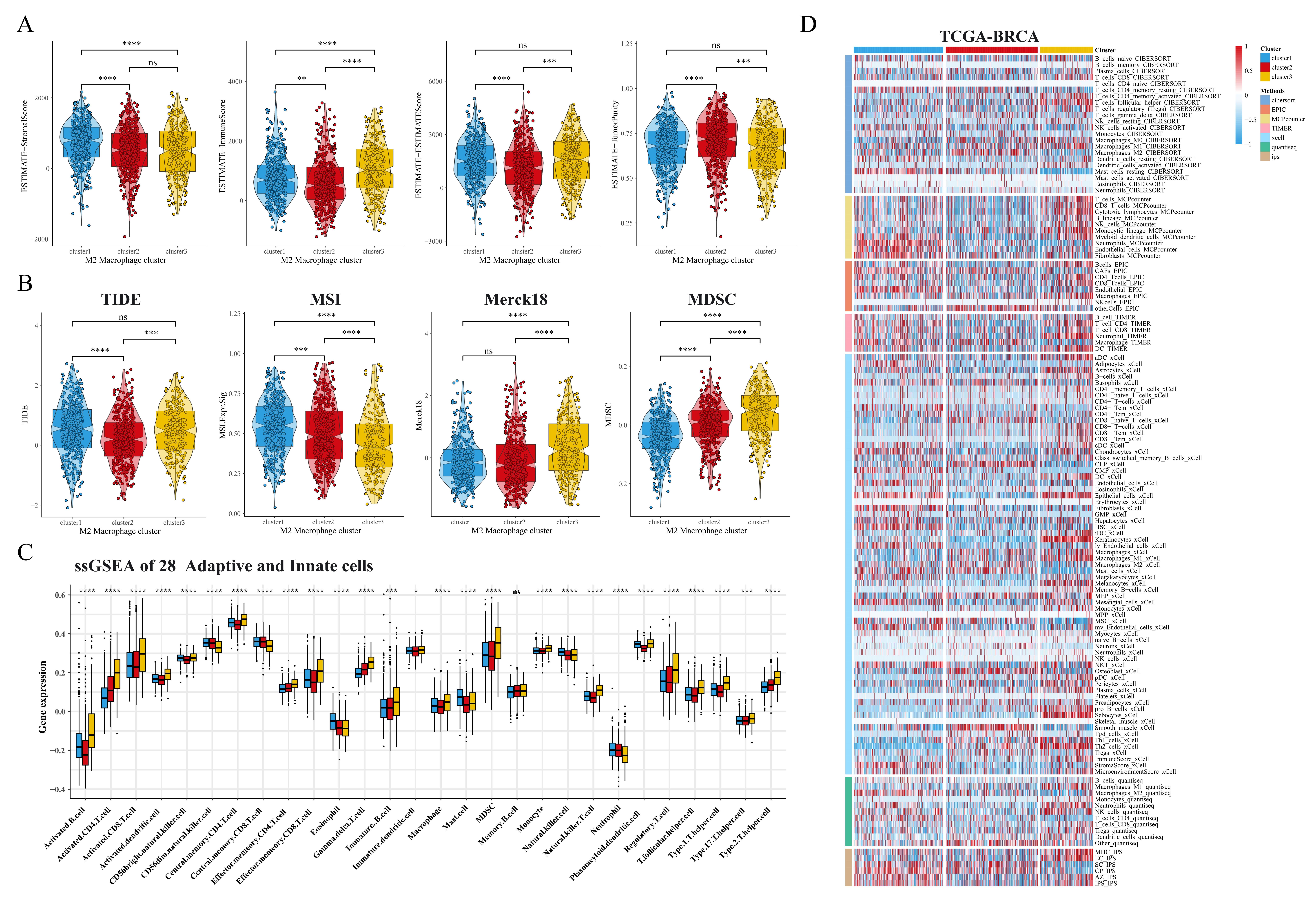


**Supplementary Figure S2.** (A) Estimate score differences between subtypes. (B) Differences in various scores from the TIDE algorithm. (C) Differences in infiltration of 28 immune cells between the three subtypes. (D) Results of multiple immune infiltration algorithms calculated by the IOBR package visualized using heatmaps.





**Supplementary Figure S3.** (A) Differences in Estimate scores between risk groups. (B) Differences in 28 immune cell infiltrations between risk groups. (C-E) Differences in immune-related gene expression (F) Comparison of immune cell infiltration between high-risk and low-risk groups. (G) Heat map showing immune cells with significant differences between risk groups. (H, I) Correlation analysis between modeled genes and risk scores and immune cells.





**Supplementary Figure S4.** (A) Heatmap showing overall (efferent and afferent) signal flow for each cell population. (B, C) Differential expression of DLG3 across staging and PAM50 subtypes. (D) Survival analysis of DLG3 in the GSE20685 cohort. (E) DLG3 expression in multiple BC monocytic cohorts. (F) High and low expression of DLG3 enriched for GSVA metabolic pathway. (G) Bar graph showing AUC values of DLG3 predicting immunotherapy response. (H) Differential expression of DLG3 in immune cells in multiple BRCA spatial transcriptome samples. (I) Differential expression of DLG3 in malignant and non-malignant regions.
